# Risk prediction in patients with heart failure with preserved ejection fraction: the LIFE-Preserved model

**DOI:** 10.1093/eurheartj/ehag182

**Published:** 2026-03-11

**Authors:** Tessa H Reitsma, Gianluigi Savarese, Łukasz Kuźma, Salil V Deo, Lisa Pennells, Steven H J Hageman, Joris Holtrop, Lina Benson, Nathalie Conrad, Lars H Lund, Anna Kurasz, Stephen Kaptoge, Spencer J Keene, Chimweta Chilala, Matilda Pitt, Robert A Fletcher, Kamlesh Khunti, Massimo Piepoli, Xavier Rossello, Jennifer S Lees, Maryam Kavousi, John William McEvoy, Angela M Wood, Rudolf A de Boer, Charlotte Andersson, Frank L J Visseren, Stefan Koudstaal, Emanuele Di Angelantonio, Jannick A N Dorresteijn, the CVD-COVID-UK/COVID-IMPACT Consortium, Emanuele Angelantonio, Ana Abreu, Frank Visseren, Maryam Kavousi, John William McEvoy

**Affiliations:** Department of Vascular Medicine and Endocrinology, University Medical Centre Utrecht, Utrecht 3508, The Netherlands; Department of Clinical Science and Education, Södersjukhuset; Karolinska Institute, Stockholm, Sweden; Department of Invasive Cardiology, Medical University of Białystok, Białystok, Poland; Department of Cardiac Surgery and Transplantology, National Medical Institute of the Ministry of Interior, Warsaw, Poland; Liverpool Centre for Cardiovascular Science, University of Liverpool, Liverpool, UK; Surgical Services, Louis Stokes Cleveland VA Medical Center, Cleveland, OH, USA; Case School of Medicine, Case Western Reserve University School of Medicine, Cleveland, OH, USA; School of Health and Wellbeing, University of Glasgow, Glasgow, UK; British Heart Foundation Cardiovascular Epidemiology Unit, Department of Public Health and Primary Care, University of Cambridge, Cambridge, UK; Victor Phillip Dahdaleh Heart and Lung Research Institute, University of Cambridge, Cambridge, UK; British Heart Foundation Centre of Research Excellence, University of Cambridge, Cambridge, UK; Department of Vascular Medicine and Endocrinology, University Medical Centre Utrecht, Utrecht 3508, The Netherlands; Department of Vascular Medicine and Endocrinology, University Medical Centre Utrecht, Utrecht 3508, The Netherlands; Department of Clinical Science and Education, Södersjukhuset; Karolinska Institute, Stockholm, Sweden; Nuffield Department of Women’s and Reproductive Health, University of Oxford, Oxford, UK; Department of Cardiovascular Sciences, KU Leuven, Leuven, Belgium; Department of Medicine and KArolinska University Hospital, Department of Cardiology, Karolinska Institutet, Stockholm, Sweden; Department of Invasive Cardiology, Medical University of Białystok, Białystok, Poland; British Heart Foundation Cardiovascular Epidemiology Unit, Department of Public Health and Primary Care, University of Cambridge, Cambridge, UK; Victor Phillip Dahdaleh Heart and Lung Research Institute, University of Cambridge, Cambridge, UK; British Heart Foundation Cardiovascular Epidemiology Unit, Department of Public Health and Primary Care, University of Cambridge, Cambridge, UK; Victor Phillip Dahdaleh Heart and Lung Research Institute, University of Cambridge, Cambridge, UK; British Heart Foundation Cardiovascular Epidemiology Unit, Department of Public Health and Primary Care, University of Cambridge, Cambridge, UK; Victor Phillip Dahdaleh Heart and Lung Research Institute, University of Cambridge, Cambridge, UK; British Heart Foundation Cardiovascular Epidemiology Unit, Department of Public Health and Primary Care, University of Cambridge, Cambridge, UK; British Heart Foundation Cardiovascular Epidemiology Unit, Department of Public Health and Primary Care, University of Cambridge, Cambridge, UK; Victor Phillip Dahdaleh Heart and Lung Research Institute, University of Cambridge, Cambridge, UK; Health Data Research UK, London, UK; The George Institute for Global Health, University of New South Wales, Sydney, Australia; Diabetes Research Centre, Leicester General Hospital, University of Leicester, Leicester, UK; Department of Biomedical Science for Health, University of Milan, Milan, Italy; Clinical University Cardiology, IRCCS Policlinico San Donato, Milan, Italy; Hospital Universitario Son Espases, Health Research Institute of the Balearic Islands, Universitat Illes Balears, Palma de Mallorca, Spain; School of Cardiovascular & Metabolic Health, University of Glasgow, Glasgow, UK; Department of Epidemiology, Erasmus MC, University Medical Centre Rotterdam, Rotterdam, The Netherlands; School of Medicine and National Institute for Prevention and Cardiovascular Health, University of Galway, Galway, Ireland; British Heart Foundation Cardiovascular Epidemiology Unit, Department of Public Health and Primary Care, University of Cambridge, Cambridge, UK; Victor Phillip Dahdaleh Heart and Lung Research Institute, University of Cambridge, Cambridge, UK; British Heart Foundation Centre of Research Excellence, University of Cambridge, Cambridge, UK; National Institute for Health and Care Research Blood and Transplant Research Unit in Donor Health and Behaviour, University of Cambridge, Cambridge, UK; Health Data Research UK Cambridge, Wellcome Genome Campus and University of Cambridge, Cambridge, UK; British Heart Foundation Data Science Centre, Health Data Research UK, London, UK; Department of Cardiology, Thorax Center, Cardiovascular Institute, Erasmus MC, Rotterdam, The Netherlands; Department of Cardiology, Copenhagen University Hospital Herlev and Gentofte, Herlev, Denmark; Center for Advanced Heart Disease, Brigham and Women’s Hospital and Harvard Medical School, Boston, MA, USA; Department of Vascular Medicine and Endocrinology, University Medical Centre Utrecht, Utrecht 3508, The Netherlands; Department of Cardiology, Thorax Center, Cardiovascular Institute, Erasmus MC, Rotterdam, The Netherlands; British Heart Foundation Cardiovascular Epidemiology Unit, Department of Public Health and Primary Care, University of Cambridge, Cambridge, UK; Victor Phillip Dahdaleh Heart and Lung Research Institute, University of Cambridge, Cambridge, UK; National Institute for Health and Care Research Blood and Transplant Research Unit in Donor Health and Behaviour, University of Cambridge, Cambridge, UK; Health Data Research UK Cambridge, Wellcome Genome Campus and University of Cambridge, Cambridge, UK; Health Data Science Research Centre, Human Technopole, Milan, Italy; Department of Vascular Medicine and Endocrinology, University Medical Centre Utrecht, Utrecht 3508, The Netherlands

**Keywords:** Risk prediction, Heart failure, HFpEF

## Abstract

**Background and Aims:**

Heart failure (HF) with preserved ejection fraction (HFpEF) constitutes a heterogeneous disease with varying prognosis. Given the rising incidence of HFpEF, accurate risk prediction for these patients is needed to identify high-risk individuals, who may benefit the most from preventive treatments. The LIFE-Preserved model was developed and validated for the prediction of individual short-term and lifetime risk for HF hospitalization or cardiovascular (CV) death in patients with HFpEF.

**Methods:**

LIFE-Preserved was derived in 20 332 patients aged 40–90 years with a left ventricular ejection fraction ≥ 50% from the Swedish HF Registry. Cause- and sex-specific Cox models were derived to predict the risk of HF hospitalization or CV death using 14 routinely available predictors. Use of age as the timescale allowed for predictions beyond the maximum follow-up duration in the derivation data, adjusted for competing risks. External validation was performed in two trials (EMPEROR-Preserved and TOPCAT-Americas) and three registries (NHS England Secure Data Environment, Veterans Affairs, and HF-Particles). Model performance was assessed by discrimination and calibration.

**Results:**

During a median follow-up of 1.8 years (interquartile range .6–4.2, maximum 19 years), 9341 first HF hospitalizations or CV deaths (46%) were observed in Swedish HF Registry. External validation included data from 28 062 patients with HFpEF [9930 (35%) first HF hospitalizations or CV deaths]. Pooled C-statistics were .714 (95% confidence interval .652–.775) in trials and .658 (95% confidence interval .599–.717 in registries, with adequate calibration in all external validation sources. Performance was similar in men and women. An interactive calculator of the LIFE-Preserved model has been made available here.

**Conclusions:**

The LIFE-Preserved model enables prediction of short-term and lifetime risk of HF hospitalization or CV death in patients with HFpEF. The model could serve as a tool to identify high-risk HFpEF patients, guiding clinical management and shared decision-making.

## Structured Graphical Abstract

**Structured Graphical Abstract:**
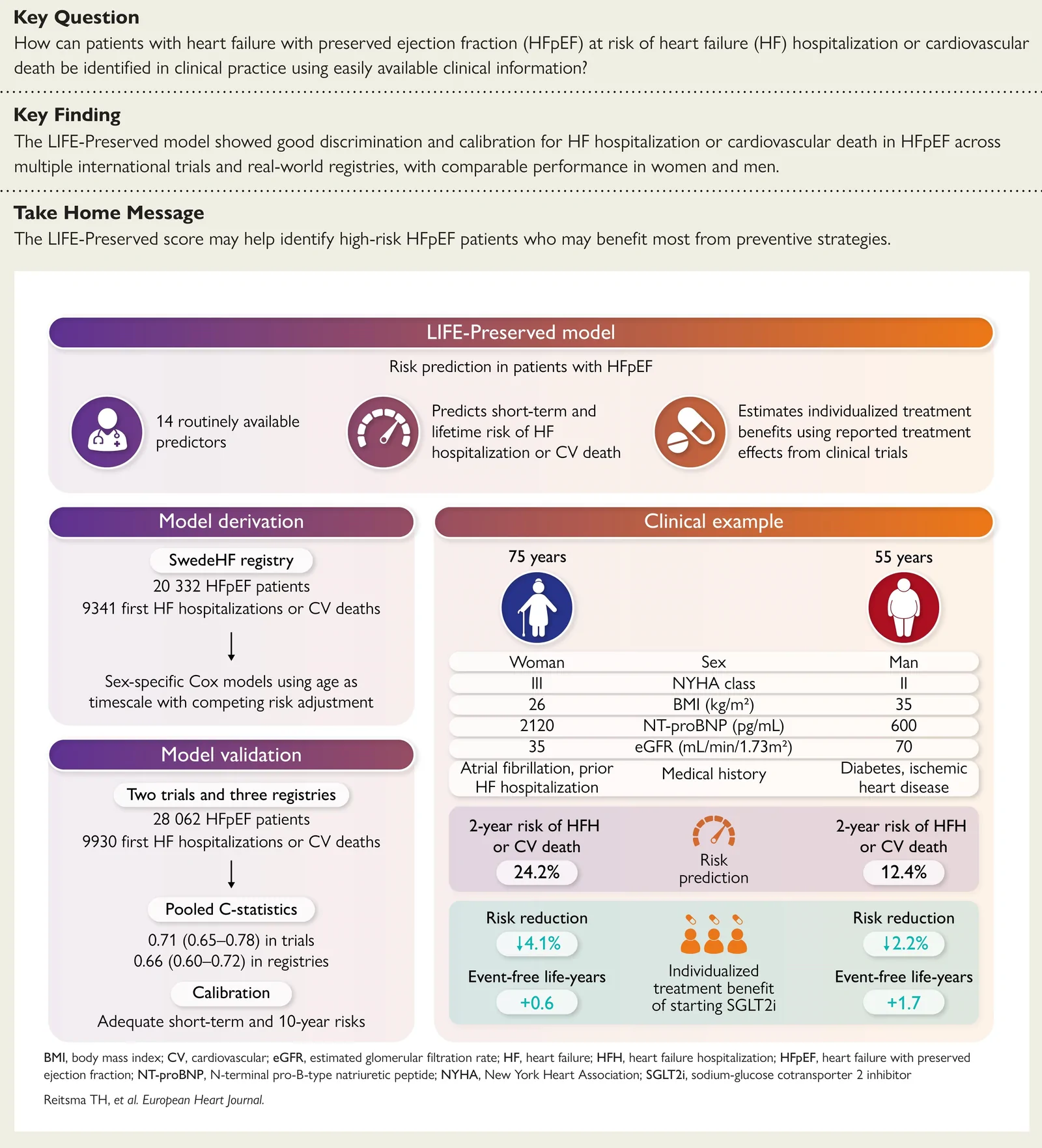
Graphical abstract of the derivation, validation, and clinical utility of the LIFE-Preserved model. For visual clarity, the clinical example illustrates a subset of predictors, but LIFE-Preserved is based on 14 routinely available predictors: age, sex, diabetes mellitus, atrial fibrillation, current smoking, prior hospitalization for heart failure (HF), chronic obstructive pulmonary disease (COPD), New York Heart Association (NYHA) class, ischaemic heart disease, N-terminal pro-B-type natriuretic peptide (NT-proBNP), body mass index (BMI), heart rate, haemoglobin, and estimated glomerular filtration rate (eGFR). CV, cardiovascular; CVD, cardiovascular death; HFH, heart failure hospitalization; HFpEF, heart failure with preserved ejection fraction; SGLT2i, sodium–glucose cotransporter 2 inhibitor.

**See the editorial comment for this article ‘Risk scores for heart failure: powerful for populations, problematic for the individual patient’, by C. Torp-Pedersen**  ***et al.*****, https://doi.org/10.1093/eurheartj/ehag183.**

## Introduction

The incidence and prevalence of heart failure (HF) with preserved ejection fraction (HFpEF) is rising globally due to the ageing population and increasing cardiometabolic risk factors.^1,2^ Heart failure with preserved ejection fraction is associated with high mortality rates, frequent hospitalizations, and significant healthcare costs.^1–3^ In recent years, new therapies have emerged that improve outcomes in patients with HFpEF.^4–7^ Sodium–glucose cotransporter 2 (SGLT2) inhibitors and the non-steroidal mineralocorticoid receptor antagonist finerenone reduce the composite risk of HF hospitalizations and cardiovascular (CV) death in patients with HFpEF.^7–9^ Semaglutide, a glucagon-like peptide-1 (GLP-1) receptor agonist, and tirzepatide, a dual GLP-1 and glucose-dependent insulinotropic polypeptide (GIP) agonist, reduce the risk of HF hospitalization and CV death in patients with obesity-related HFpEF.^10,11^ However, patients with HFpEF have highly heterogeneous clinical profiles with varying prognosis, and absolute benefit of treatment options may vary greatly between patients.^12^ In fact, some patients are at relatively low risk, resulting in reduced absolute risk reductions, a higher number needed to treat (NNT), and potentially lower cost-effectiveness.^8^ This highlights the need for accurate risk prediction to optimize treatment allocation to patients most likely to benefit. In addition, risk prediction may facilitate shared decision-making and increase motivation for drug treatment and lifestyle changes.^13^

Previous prediction models for the HFpEF population are restricted to predict 1- or 2-year risks.^14–19^ However, as HFpEF is a chronic disease requiring lifelong treatment, prediction models should ideally provide long-term or lifetime risk to align with the clinical decision-making horizon.^20^ Additionally, prior models were not HFpEF specific or were derived only in trial populations, which are known to suffer from healthy participant bias. Therefore, to ensure clinical applicability, validation in real-world population-based data is required. The European Society of Cardiology (ESC) CV disease prevention guidelines recommend the use of 10-year and lifetime risk predictions to guide clinical decision-making.^21–25^ For patients with HF and reduced ejection fraction (HFrEF), the LIFE-HF model is an externally validated risk prediction model for the estimation of lifetime risk of HF hospitalization or CV death.^26^ A similar model for patients with HFpEF could help to improve HFpEF management by supporting personalized medicine and shared decision-making.

The objective of the present study was to develop and externally validate the LIFE-Preserved model for individual prediction of short-term and lifetime risk of HF hospitalization or CV death in patients with HFpEF.

## Methods

### Study populations

The LIFE-Preserved risk model was developed in HFpEF patients from the Swedish HF Registry (SwedeHF). Details on SwedeHF have been published previously.^27^ Briefly, SwedeHF is an ongoing nationwide registry in Sweden founded in 2000, with nationwide coverage since 2003. Around 85% of all hospitals in Sweden actively participate in the registry, and in 2020, ∼31% of all prevalent HF patients in Sweden were registered.^28,29^ The Swedish HF Registry includes patients based on a clinician-judged diagnosis of HF and, since 2017, also identifies patients through the National Patient Register using International Classification of Diseases 10th revision (ICD-10) codes (I50.0, I50.1, I50.9, I42.0, I42.6, I42.7, I25.5, I11.0, I13.0, I13.2). Patients can be included from the outpatient and the inpatient setting in SwedeHF. Baseline characteristics are recorded upon inclusion. Information regarding medical history and other variables (e.g. comorbidities, date and cause of death, hospitalizations, socioeconomic status, and prescriptions) can be retrieved through linkage with the Swedish National Patient Register, Cause of Death Register, Statistics Sweden, and National Prescribed Drug Register (see Supplementary data online, *Table S1*). Linkage is achieved though the personal unique identification number that all the residents in Sweden have.

For the current study, patients aged between 40 and 90 years, with a left ventricular ejection fraction (LVEF) ≥ 50% at baseline and no earlier registration with documented LVEF < 50%, were eligible for analyses. Only those patients with a registration in SwedeHF between 1 January 2006 and 1 February 2020 were included to eliminate the potential impact of the COVID-19 pandemic on study results, and follow-up was truncated on 1 February 2020 for the same reason. If a patient had multiple eligible registrations, the first one was selected (see Supplementary data online, *Figure S1*, for the flowchart of patient selection).

External validation was performed in HFpEF (LVEF ≥ 50%) patients using population-based data from three registries, the HF-Particles cohort in Poland,^30^ the Department of Veterans Affairs (VA) cohort in the USA,^31^ and the NHS England Secure Data Environment (NHSE SDE) through the British Heart Foundation Data Science Centre CVD-COVID-UK/COVID-IMPACT Consortium.^3^ In addition, external validation was performed in HFpEF (LVEF ≥ 50%) patients using data from two trials, the EMPEROR-Preserved trial^7^ and TOPCAT-Americas trial.^32^ Patients aged 40–89 years were included in the external validation analyses, as the LIFE-Preserved model estimates risks up to age 90, allowing 1-year risk prediction for individuals aged 89 years. Detailed descriptions of the external validation data sources can be found in the *Supplementary Methods*.

### Outcomes and predictors

The primary outcome was a composite of HF hospitalization or CV death, which was assessed with a time-to-first-event analysis. This endpoint was chosen because it represents a clinically actionable outcome in HFpEF, directly targeted by contemporary preventive therapies, and therefore most relevant for informing risk stratification and clinical decision-making. Non-CV death was used as the competing outcome.

In SwedeHF, hospitalizations were extracted from the National Patient Register, and HF hospitalization was defined as the first hospitalization with HF registered as the primary diagnosis during follow-up. Cardiovascular death was defined as death with CV disease registered as the primary cause of death in the Cause of Death Register. All deaths that were not classified as CV deaths were defined as non-CV deaths. In external validation, outcomes were assessed by linkage to hospital records and mortality registries (the NHSE SDE, HF-Particles, and VA) or adjudicated by an endpoint committee in clinical trials (EMPEROR-Preserved and TOPCAT-Americas). Full details on outcome definitions and the corresponding ICD-10 codes for all data sources are described in Supplementary data online, *Table S2*.

Candidate predictors of the LIFE-Preserved model were based on a literature review of prior risk models (see *Supplementary Methods* and Supplementary data online, *Figure S2*).^14–19^ Candidate predictors were diabetes mellitus, current smoking, any previous hospitalization for HF, chronic obstructive pulmonary disease, New York Heart Association (NYHA) class, ischaemic heart disease, N-terminal pro-B-type natriuretic peptide (NT-proBNP), body mass index (BMI), heart rate, haemoglobin, and estimated glomerular filtration rate (eGFR) based on serum creatinine calculated with the 2009 Chronic Kidney Disease Epidemiology Collaboration formula^33^ (see Supplementary data online, *Table S3*). Additionally, an expert panel reviewed predictors based on clinical relevance and availability for HFpEF, and five additional predictors were identified for consideration: atrial fibrillation, cerebrovascular disease (stroke or transient ischaemic attack), loop diuretic use, systolic blood pressure, and duration of HF. Of these, only history of atrial fibrillation showed a statistically significant improvement of internal C-statistics for the primary outcome of HF hospitalization or CV death compared with the core model and was therefore included in the final model (see Supplementary data online, *Figure S3*). The same predictors were used for the primary and the competing outcomes.

Missing data were handled with imputation, using predictive mean matching for continuous variables and logistic regression for binary variables. For predictor variables with missing data in SwedeHF, values were imputed using two separate sex-specific multiple imputation by chained equation models with 45 imputed datasets and 45 iterations, approximately the maximum predictor missingness (see Supplementary data online, *Table S4*, and details in *Supplementary Methods*). Convergence of the imputation models was checked by inspecting trace plots. Missing predictor values in the external validation data were handled within each data source separately (see *Supplementary Methods*).

### Model development

To allow for sex differences in the relative effects of predictors, cause-specific Cox proportional hazards models were derived for men and women separately. For each sex, two separate cause-specific Cox proportional hazards models were constructed for prediction of first hospitalization for HF or CV death and prediction of non-CV death as a competing outcome. Adjusting for competing events is necessary for accurate risk prediction in a high-risk population to avoid overestimation of risk estimates.^34^ Age was used as the time scale with age at inclusion defined as the start of follow-up (left truncation) and age at event or censoring defined as the end of follow-up (right censoring). This allows for estimation of risks over the age range of the entire population, enabling estimation risk at any time horizon as well as lifetime risk (i.e. risk until age 90).^35–37^ To account for the high readmission risk observed in recently hospitalized HFpEF patients, a time-varying first-year risk adjustment was applied to patients with a recent HF hospitalization (within 6 months prior to inclusion).^38^ Continuous predictors were truncated at the 1st and 99th percentiles to limit outlier effects, and non-linear transformations were applied if they improved model fit based on Akaike’s information criterion (AIC); details are provided in *Supplementary Methods*. Age interactions were considered when the proportional hazards assumption was violated, as assessed by visual inspection of plotted Schoenfeld residuals. As SGLT2 inhibitors and GLP-1 receptor agonists were hardly used during the study period (<1% use at baseline), these were not accounted for in model development.

Sex-specific baseline survival probabilities were derived for HF hospitalization or CV death and non-CV death by estimating 3-month interval survival over the complete age range (40–90 years). All baseline survival probabilities were estimated for a reference patient and smoothed using non-linear functions weighted by the number of patients in each age interval (see *Supplementary Methods*).

### Individual risk prediction

For each patient, the risk of HF hospitalization or CV death and non-CV death was estimated for every remaining 3-month interval up to age 90 using validated lifetable methods.^35–37^ This approach, which has been shown reliable for prediction horizons of at least 17 years,^35^ provides interval-specific survival probabilities for both outcomes. These were combined to calculate the probability of surviving each interval free of events, accounting for competing risks. These interval survival probabilities were multiplied to calculate the cumulative probability of reaching a specific age free of any event. Heart failure hospitalization-free life expectancy was defined as the age at which this cumulative survival probability drops below 50%. Lifetime risks for each outcome were defined as the cumulative risk from the current age of the patient until the maximum age of 90 years. Detailed descriptions of lifetable estimations are provided in the *Supplementary Methods*.

### External validation

Performance of the LIFE-Preserved model was assessed by estimating Harrell's C-statistic for discrimination and calibration plots of the predicted vs observed risk for calibration. All were adjusted for competing risks.^39^ To ensure stable estimates, external validation was performed on the last full year in which at least 75th percent was still in follow-up in the respective data source, which was at 2 years for EMPEROR-Preserved (*n* = 3928) and NHSE SDE (*n* = 15 795), 4 years for TOPCAT-Americas (*n* = 1495), and 8 years for HF-Particles (*n* = 838). In the VA, which was the largest cohort with long-term follow-up, external validation was performed at 10 years (*n* = 4499). C-statistics were pooled using inverse-variance weighting,^40^ which was performed separately for trials (EMPEROR-Preserved and TOPCAT-Americas) and registries (NHSE SDE, HF-Particles, and VA), given the differences in HFpEF phenotyping and characteristics between trials and registries. C-statistics were compared with the PREDICT-HFpEF model^19^ in EMPEROR-Preserved. In all external validation data, model performance was assessed in men and women separately. Additionally, to account for potential geographical differences in average risk and study-specific selection mechanisms, the model was recalibrated using the expected–observed (EO) ratio (a single multiplicative constant) when necessary (see *Supplementary Methods* for details).

### Sensitivity analyses

In EMPEROR-Preserved, model performance was assessed in subgroups of treatment arm (SGLT2 inhibitor or placebo) and stratified by diabetes status and obesity (BMI ≥ 30 kg/m^2^). In the NHSE SDE, a sensitivity analysis was conducted during the COVID-19 period (follow-up from 1 January 2020 to 1 March 2022). Detailed description of this COVID cohort can be found in the *Supplementary Methods*.

### Prediction of individualized treatment benefit

A potential application of the LIFE-Preserved model is the estimation of individualized benefits from starting preventive treatment, as has been done in previous risk models.^35–37,41^ The LIFE-Preserved model can be combined with the latest relative treatment effects from trials and meta-analyses to estimate absolute individualized treatment effects, for which a detailed approach is available in *Supplementary Methods*. The individualized treatment benefits can be expressed as absolute risk reduction or gain in HF hospitalization-free life years. If a patient is already receiving one or more evidence-based treatments, the corresponding treatment effects can be combined to LIFE-Preserved in the same way, providing risk estimates that reflect the patient’s current treatment regimen. For illustration, the individualized treatment benefit of starting a SGLT2 inhibitor [hazard ratio (HR) .81]^42^ was estimated for two example patients. Suggested HRs for treatment options are reported in Supplementary data online, *Table S5*, and serve as illustrative examples derived from current evidence. These should be updated as new treatments and trial results become available.

All analyses were conducted using R-statistical programming (version 4.4.1, R Foundation for Statistical Computing, Vienna, Austria). Results were reported in line with the TRIPOD statement (checklist in *Supplementary Material*).^43^

## Results

### Baseline characteristics

The SwedeHF population used for derivation consisted of 10 481 women and 9851 men, with a median age at baseline of 80 years [interquartile range (IQR) 74–85] and 77 years (69–83), respectively. Detailed sex-specific baseline characteristics are presented in *Table 1* and in Supplementary data online, *Table S6*, for baseline characteristics stratified for in- and outpatients. In women, 5044 (48%) first HF hospitalizations or CV deaths occurred during a median follow-up of 1.7 years (IQR .5–4.2 years), and 3175 (30%) non-CV deaths occurred over a total median follow-up of 2.7 years (IQR 1.0–5.3). In men, 4297 (44%) first HF hospitalizations or CV deaths occurred during a median follow-up of 1.8 years (IQR .6–4.2), and 2866 (29%) non-CV deaths occurred over a total median follow-up of 2.6 years (IQR 1.0–5.3). Missingness was high for some predictors [NT-proBNP (45%), BMI (35%), NYHA class (33%), and current smoking (29%)] and were below 6% for other predictors (see Supplementary data online, *Table S4*). Because complete case analysis may lead to loss of statistical power and possible bias, values were imputed using sex-specific multiple imputation by chained equation models.^44^ Assessment of trace plots demonstrated good convergence and stable parameter estimation across the 45 iterations of the multiple imputation models.

**Table 1:** Sex-specific baseline characteristics of SwedeHF

	Women (*n* = 10 481)	Men (*n* = 9851)
Age (years)	80 [74–85]	77 [69–83]
Current smokers	867 (8)	891 (9)
Physical signs		
Body mass index (kg/m^2^)	27 [23–32]	27 [24–31]
Systolic blood pressure (mmHg)	130 [120–148]	130 [120–145]
Diastolic blood pressure (mmHg)	70 [65–80]	73 [65–80]
Heart rate (b.p.m.)	72 [64–83]	70 [61–80]
Clinical features of HF		
NYHA class		
I	1390 (13)	1685 (17)
II	4809 (46)	4549 (46)
III	3897 (37)	3291 (33)
IV	385 (3.7)	326 (3.3)
Duration of HF (years)	.2 [0–2.6]	.2 [0–2.8]
Left bundle branch block	620 (6)	732 (7)
Medical history		
Prior HFH	5833 (56)	5388 (55)
Recent HFH (≤6 months)	4829 (46)	4202 (43)
Diabetes mellitus	2752 (26)	2989 (30)
Ischaemic heart disease	4494 (43)	4866 (49)
Chronic obstructive pulmonary disease	1739 (17)	1572 (16)
Obesity^a^	3492 (33)	3032 (31)
Atrial fibrillation	6665 (64)	6435 (65)
Stroke or transient ischaemic attack	1875 (18)	1809 (18)
Peripheral artery disease	858 (8)	1046 (11)
Valve disease	3662 (35)	3136 (32)
Dilated cardiomyopathy	271 (3)	455 (5)
Laboratory measurements		
NT-proBNP (pg/mL)	2120 [972–4343]	1755 [722–3700]
Haemoglobin (mmol/L)	7.8 [7.1–8.4]	8.2 [7.3–8.9]
Estimated GFR (mL/min/1.73 m^2^)	55 [40–71]	46 [33–59]
Medication use		
Loop diuretics	8552 (82)	7578 (77)
ACE-inhibitor/ARB	7404 (71)	7364 (75)
Beta-blocker	8583 (82)	7897 (80)
Statin	4010 (38)	4779 (48)
MRA	3164 (30)	2937 (30)
Digoxin	1739 (17)	1125 (11)
Nitrate	1624 (16)	1333 (14)
Device therapy^b^	134 (1)	266 (3)
ARNI	24 (.2)	44 (.4)
SGLT2 inhibitor	25 (.2)	68 (.7)
GLP-1 receptor agonist	44 (.4)	79 (.8)
Outcomes: incidence (%) | event rate per 1000 PY		
First HFH/CV death	5044 (48) | 169	4297 (44) | 148
First HFH	3735 (36) | 125	3226 (33) | 111
Total number of HFH	9158 (36) | 307	8575 (33) | 296
Non-CV death	3175 (30) | 83	2866 (29) | 80
CV death	2776 (26) | 73	2293 (23) | 64
Follow-up time for first HFH (years)	1.7 [.5–4.2]	1.8 [.6–4.2]
Follow-up time for death (years)	2.7 [1.0–5.3]	2.6 [1.0–5.3]

Data are presented as *n* (%) or median (25th–75th percentile).

HF, heart failure; NYHA, New York Heart Association; NT-proBNP, N-terminal pro-B-type natriuretic peptide; GFR, glomerular filtration rate (calculated with the 2009 Chronic Kidney Disease Epidemiology Collaboration formula); ACE, angiotensin-converting enzyme; ARB, angiotensin receptor blocker; ARNI, angiotensin receptor–neprilysin inhibitor; MRA, mineralocorticoid receptor antagonist; SGLT2, sodium–glucose cotransporter 2; GLP-1, glucagon-like peptide-1; HFH, heart failure hospitalization; CV, cardiovascular; PY, patient-years.

^a^Obesity is defined as body mass index of ≥30 kg/m^2^.

^b^Device therapy includes cardiac resynchronization therapy and implantable cardioverter-defibrillator.

### Model derivation

The cause-specific HRs of the LIFE-Preserved model are presented in *Table 2*. For interpretability, Supplementary data online, *Figure S4*, illustrates the effect of continuous predictors and age interactions on the primary outcome of HF hospitalization or CV death across the full range of predictor values and ages. All parameters needed for individual predictions are shown in the Supplementary data; age-specific baseline survivals are presented in Supplementary data online, *Table S7*, and Supplementary data online, *Figure S5*, and equations for linear predictors are provided in Supplementary data online, *Table S8*, detailed explanation is available in *Supplementary Methods*. Internal validation C-statistic was .732 [95% confidence interval (CI) .723–.741] for the primary outcome of HF hospitalization or CV death. Discrimination C-statistics were similar in men and women and higher in outpatients compared with those included from the inpatient setting (see Supplementary data online, *Table S9*). Predicted risks matched observed incidence in internal validation for both outcomes at 2 and 10 years, and this alignment was maintained when stratified for in- and outpatients (see Supplementary data online, *Figure S6*).

**Table 2:** Hazard ratios of the predictors from the LIFE-Preserved model

	Hazard ratios (95% CI)			
	HF hospitalization or CV death		Non-CV death	
	Women	Men	Women	Men
Age at baseline (per 10 years)	1.61 (1.38–1.89)	1.58 (1.35–1.86)	.73 (.63–.86)	.71 (.60–.83)
Current smoking (vs never/former)	1.16 (1.02–1.32)	1.19 (1.04–1.36)	.96 (.82–1.12)	1.30 (1.11–1.52)
NYHA class III/IV (vs I/II)	1.24 (1.16–1.34)	1.34 (1.24–1.44)	1.37 (1.26–1.50)	1.29 (1.17–1.43)
Ischaemic heart disease	1.20 (1.13–1.27)	1.15 (1.08–1.23)	.86 (.80–.93)	.86 (.80–.93)
Chronic obstructive pulmonary disease	1.30 (1.20–1.41)	1.33 (1.22–1.44)	2.05 (1.88–2.25)	1.76 (1.60–1.93)
Atrial fibrillation	1.21 (1.13–1.29)	1.16 (1.08–1.25)	.86 (.79–.93)	.90 (.82–.98)
Prior HF hospitalization (at any time)	1.38 (1.28–1.48)	1.38 (1.28–1.49)	1.14 (1.06–1.24)	1.23 (1.13–1.33)
HF hospitalization in past 6 months^b^	2.06 (1.91–2.23)	1.97 (1.81–2.14)	1.26 (1.13–1.41)	1.26 (1.12–1.41)
Diabetes mellitus	1.53 (1.38–1.69)^a^	1.39 (1.28–1.52)^a^	1.35 (1.32–1.38)	1.26 (1.23–1.28)
NT-proBNP (4000 vs 800 pg/mL)^c^	1.47 (1.38–1.57)	1.50 (1.40–1.61)	1.44 (1.33–1.57)	1.33 (1.22–1.46)
BMI (35 vs 25 kg/m^2^)^d^	1.14 (1.05–1.23)	1.24 (1.11–1.38)	.88 (.80–.98)	.94 (.82–1.06)
Haemoglobin (7 vs 8.5 mmol/L)^e^	1.10 (1.04–1.15)	1.13 (1.08–1.19)	1.21 (1.14–1.28)	1.28 (1.20–1.35)
Estimated GFR (35 vs 65 mL/min/1.73 m^2^)^e^	1.24 (1.17–1.31)	1.17 (1.10–1.25)	1.29 (1.21–1.38)	1.12 (1.04–1.20)
Heart rate (80 vs 60 b.p.m.)^e^	1.06 (1.00–1.12)	1.14 (1.08–1.21)	1.22 (1.13–1.31)	1.18 (1.11–1.27)

The presented hazard ratios are equal to the exponential of the coefficients, and they cannot be causally interpreted. Because age was used as the underlying time scale, part of its predictive effect is captured by the baseline hazard, which limits the clinical interpretability of its hazard ratio. Hazard ratios for continuous predictors are shown for clinically interpretable comparisons; for insight in the detailed relationship of the continuous predictors and age interactions, see Supplementary data online, *Figure S4*.

CI, confidence interval; HR, hazard ratio; NYHA, New York Heart Association; HF, heart failure; NT-proBNP, N-terminal pro-B-type natriuretic peptide; BMI, body mass index; eGFR, estimated glomerular filtration rate (calculated with the 2009 Chronic Kidney Disease Epidemiology Collaboration formula).

^a^Interaction with age, hazard ratio shown for age 70 years.

^b^Time-varying effect, only applies to the first year of risk predictions and is on top of the effect of prior heart failure hospitalization at any time.

^c^Log-transformation.

^d^Cubic transformation.

^e^Squared transformation.

### External validation

External validation of the LIFE-Preserved risk model included data from 28 062 patients with HFpEF in which 9930 (35%) first HF hospitalizations or CV deaths were observed. Median follow-up times ranged from 1.9 years (IQR .6–2.8) in the NHSE SDE to 6.8 years (IQR 4.2–8.6) in HF-Particles. Detailed patient characteristics of the external validation data sources are presented in *Table 3*.

**Table 3:** Baseline characteristics in external validation data

	Trials		Registries		
	EMPEROR-Preserved (*n* = 3955)	TOPCAT-Americas (*n* = 1553)	NHSE SDE (*n* = 17 005)	HF-Particles (*n* = 907)	VA (*n* = 4642)
Age (years)	74 [67–79]	73 [64–79]	79 [72–85]	71 [63–77]	66 [62–71]
Women	1988 (50)	802 (52)	9290 (55)	457 (50)	62 (1)
Current smokers	248 (6)	90 (6)	1035 (6)	16 (2)	935 (20)
Physical signs					
Body mass index (kg/m^2^)	30 [26–34]	33 [28–39]	30 [26–35]	28 [25–32]	31 [28–35]
Heart rate (b.p.m.)	69 [61–78]	68 [61–76]	74 [65–84]	78 [70–84]	^a^
Clinical features of HF					
NYHA class (III/IV)	736 (19)	556 (36)	12 190 (72)	355 (39)	1727 (37)
Medical history					
Prior HFH	871 (22)	899 (58)	17 005 (100)	82 (9)	983 (21)
Recent HFH (<6 months)	871 (22)	239 (15)	7145 (42)	67 (7)	769 (17)
Diabetes mellitus	1896 (48)	692 (45)	7915 (47)	266 (29)	2447 (53)
Ischaemic heart disease	1125 (28)	699 (45)	10 575 (62)	359 (40)	4642 (100)
Chronic obstructive pulmonary disease	537 (14)	252 (16)	5740 (34)	43 (5)	1101 (24)
Atrial fibrillation	2147 (54)	643 (41)	11 525 (68)	311 (34)	358 (8)
Laboratory measurements					
NT-proBNP (pg/mL)	941 [478–1672]	738 [432–1393]	3717 [1549–6749]	^a^	^a^
Haemoglobin (mmol/L)	8.2 [7.6–8.8]	7.9 [7.3–8.7]	7.3 [6.4–8.1]	8.2 [7.6–8.8]	8.4 [7.6–9.1]
Estimated GFR (mL/min/1.73 m^2^)	59 [45–74]	58 [47–73]	51 [37–68]	83 [65–93]	73 [58–90]
Follow-up time (years)	2.0 [1.4–2.7]	2.5 [1.4–3.9]	1.9 [.6–2.8]	6.8 [4.2–8.6]	3.3 [.9–6.3]
Incidence of HFH/CV death	579 (15)	451 (29)	6810 (40)	341 (38)	1749 (38)
Event rate of HFH/CV death per 1000 PY	73	110	241	60	98
Event rate of non-CV death per 1000 PY	25	29	137	19	38

Data are presented as *n* (%) or median (25th–75th percentile).

HF, heart failure; NYHA, New York Heart Association; NT-proBNP, N-terminal pro-B-type natriuretic peptide; eGFR, estimated glomerular filtration rate (calculated with the 2009 Chronic Kidney Disease Epidemiology Collaboration formula); HFH, heart failure hospitalization; CV, cardiovascular; PY, patient-years.

^a^Data for this variable are missing completely in this data source.

**Figure 1:**
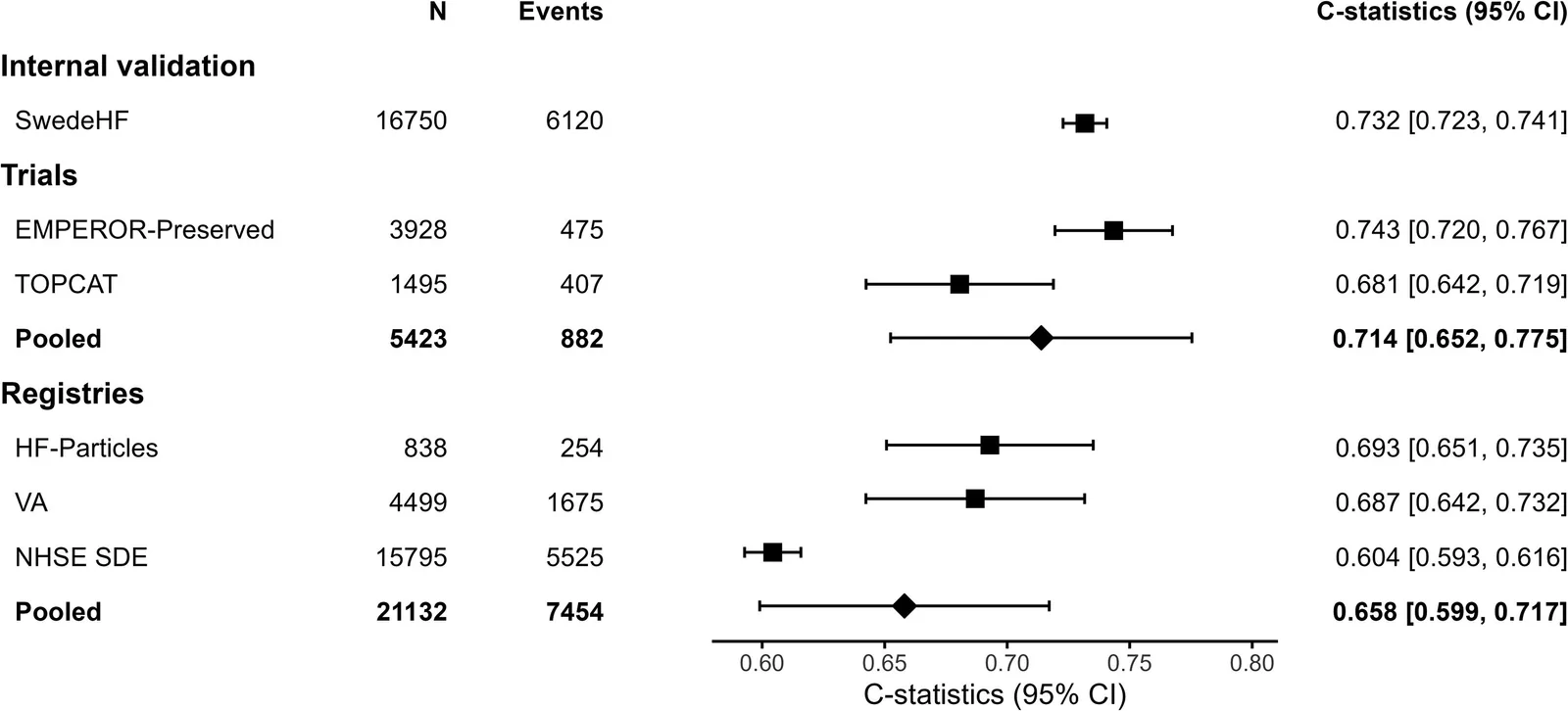
C-statistics for heart failure hospitalization or cardiovascular death in external validation data. Forest plot displaying the discrimination in all external validation data is based on Harrell’s C-statistic for heart failure hospitalization or cardiovascular death adjusted for non-cardiovascular death. Discrimination was assessed at the 75th percentile of follow-up duration (2-year risks for EMPEROR-Preserved and the NHSE SDE, 4-year risks for TOPCAT-Americas, and 8-year risks for HF-Particles) and at 10-year risks for the Department of Veterans Affairs. Pooled C-statistics were calculated using inverse-variance weighting. Cohort sizes here may be lower than the total cohort population listed in *Table 3* as risks can only be estimated up to age 90 (e.g. 89-year-olds can have 1-year risk but no risks for 2-year or higher and are consequently excluded at the horizon for validation). CI, confidence interval

For the primary outcome of HF hospitalization and CV death, pooled C-statistics were .714 (95% CI .652–.775) in trials and .658 (.599–.717) in registries (*Figure 1*). Model discrimination remained consistent when validated at a prediction horizon of 8 years in HF-Particles (C-statistic .693, 95% CI .651–.735) and 10 years in VA (C-statistic .687, 95% CI .642–.732). C-statistics were similar in men and women (see Supplementary data online, *Figure S7*) and higher compared with the existing PREDICT-HFpEF model^19^ in EMPEROR-Preserved (see Supplementary data online, *Table S10*). For the competing outcome of non-CV death, C-statistics were consistent in external validation (see Supplementary data online, *Table S9*).

**Figure 2:**
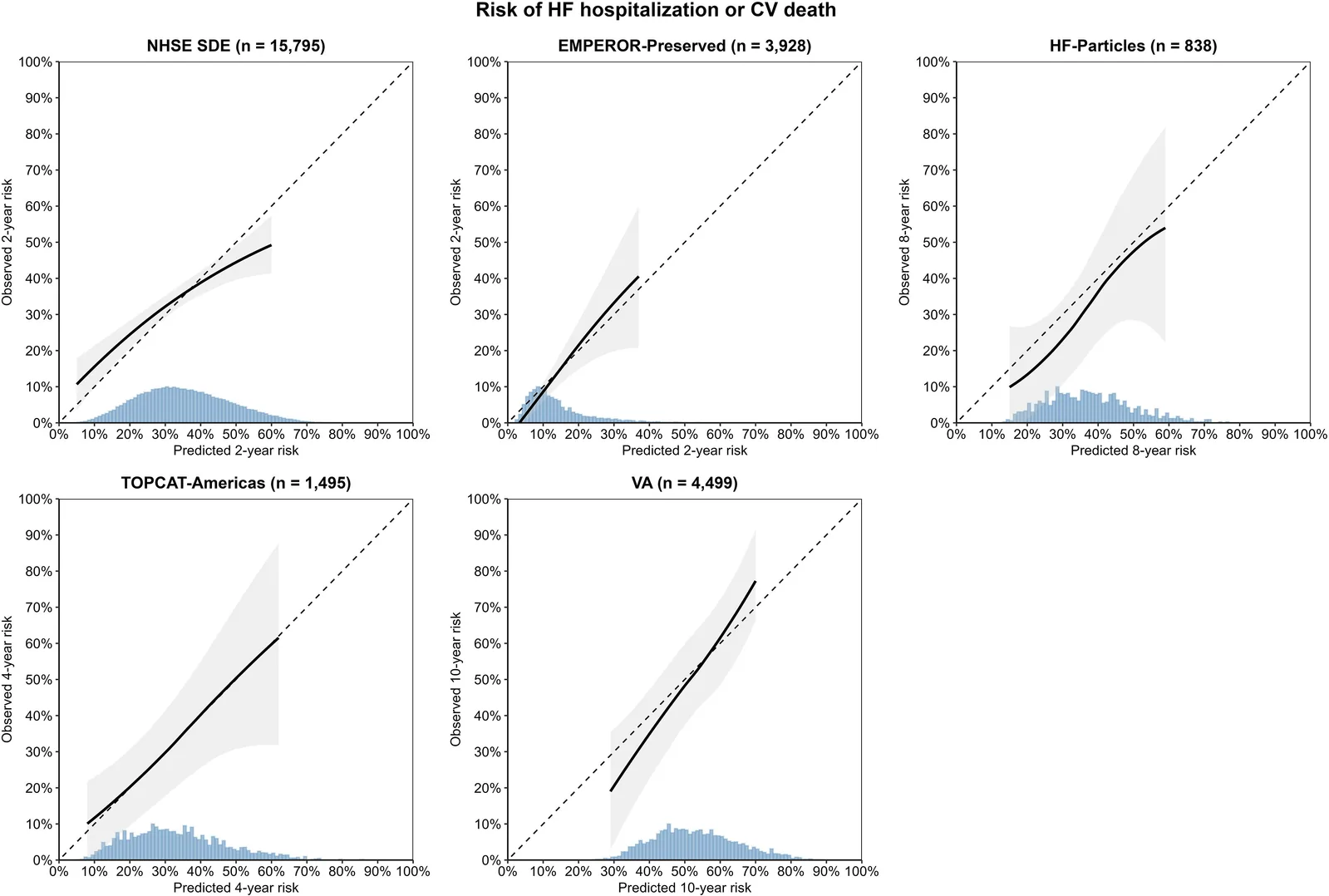
Calibration for heart failure hospitalization or cardiovascular death in external validation data. Calibration of the LIFE-Preserved model in external validation. Risks were recalibrated in the NHSE SDE and EMPEROR-Preserved using expected–observed ratios specified in Supplementary data online, *Table S11*. Smoothed calibration plots of predicted risks vs observed risks and a histogram of the predicted risks is shown. Cohort sizes here may be lower than the total cohort population listed in *Table 2* as risks can only be estimated up to age 90 (e.g. 89-year-olds can have 1-year risk but no risks for 2-year or higher and are consequently excluded at the horizon for validation). HF, heart failure; CV, cardiovascular

Predicted risks of HF hospitalization or CV death from the LIFE-Preserved model were in line with the observed incidence in VA, TOPCAT-Americas, and HF-Particles (*Figure 2*). In EMPEROR-Preserved (EO ratio 1.55 for women and 1.31 for men) and the NHSE SDE (EO ratio 1.24 for women and 1.28 for men), some overestimation was observed. Expected–observed ratios in all external validation cohorts are shown in Supplementary data online, *Table S11*. Following recalibration, predicted risks of HF hospitalization or CV death were aligned with observed incidence in all validation data sources (*Figure 2*) and similar in men and women (see Supplementary data online, *Figures S8*–*S9*). The risk of non-CV death was overestimated prior to recalibration in the external trial populations and underestimated in the NHSE SDE (see Supplementary data online, *Figure S10* and *Table S11*).

### Sensitivity analyses

In EMPEROR-Preserved, performance was similar in subgroups of diabetes status and obesity regarding discrimination (Supplementary data online, *Table S9*) and calibration (see Supplementary data online, *Figure S11*). After accounting for the treatment effect of the intervention as reported in the EMPEROR-Preserved trial (HR .79),^7^ calibration (see Supplementary data online, *Figure S11*) and discrimination (see Supplementary data online, *Table S9*) were similar in the treatment and placebo groups. In a sensitivity analysis of the NHSE SDE comparing the COVID-19 pandemic period with the post-COVID-19 pandemic period (main analysis for the NHSE SDE), similar calibration results were observed (see Supplementary data online, *Figure S12*).

### Prediction of individualized treatment benefit

**Figure 3:**
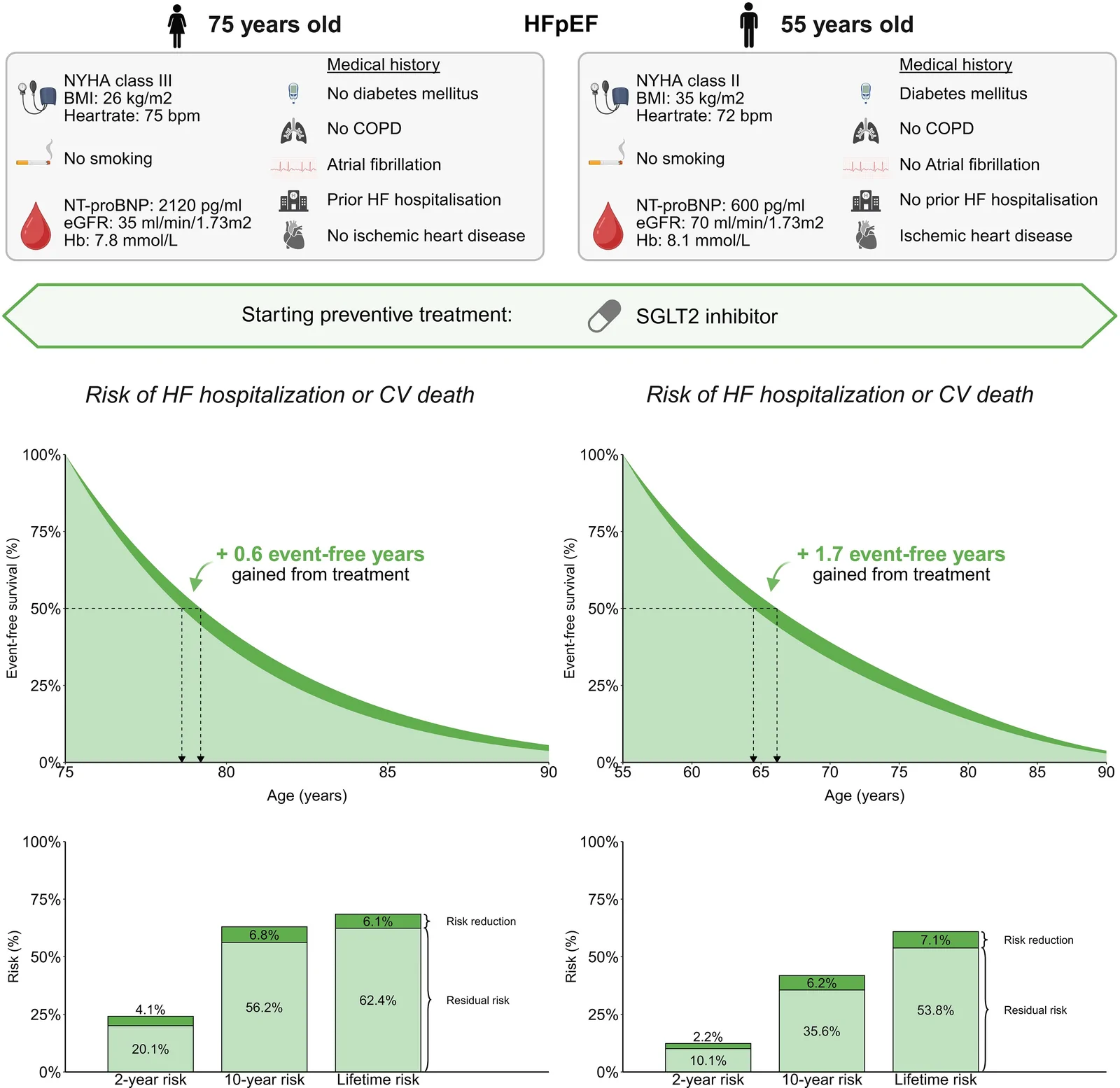
Clinical example of estimation of individualized treatment benefit. Clinical example of estimation of individual short-term and lifetime risk and individualized treatment benefit after starting a sodium–glucose cotransporter 2 inhibitor for two different heart failure with preserved ejection fraction patients. Event-free survival in the curves is defined as survival without any heart failure hospitalization or cardiovascular death. Treatment benefits were estimated by combining the individual absolute risks from the LIFE-Preserved model with the published hazard ratios from trials or meta-analyses. In this example, the published hazard ratio of .81 was used as treatment effect of starting a sodium–glucose cotransporter 2 inhibitor.^41^ Treatment benefits do not reflect observed treatment use in the cohorts and should be interpreted as projections of potential benefit. The individualized treatment benefit can be shown as an absolute risk reduction (bar chart, bottom row) or as gain in heart failure hospitalization-free life-years (darker area in survival plot, top row). HFpEF, heart failure with preserved ejection fraction; NYHA, New York Heart Association; BMI, body mass index; b.p.m., beats per minute; NT-proBNP, N-terminal pro-B-type natriuretic peptide; eGFR, estimated glomerular filtration rate; Hb, haemoglobin; COPD, chronic obstructive pulmonary disease; HF, heart failure; CV, cardiovascular; SGLT2, sodium–glucose cotransporter 2. Created in https://BioRender.com

A clinical example of the possible use of the LIFE-Preserved model is illustrated in *Figure 3*. The LIFE-Preserved model can be used to predict the individual 2-year, 10-year, and lifetime risks of HF hospitalization or CV death for two example HFpEF patients: a 75-year-old woman and a 55-year-old man. Combined with HRs from a meta-analysis,^41^ individualized treatment benefits for starting a SGLT2 inhibitor can be estimated for the two example patients. Risk factor levels of both example patients at baseline are presented in *Figure 3*. The treatment benefit can be expressed as absolute risk reduction, for example, the reduction in 2- or 10-year risk, or as gain in HF hospitalization-free life years (*Figure 3*). The gain in HF hospitalization-free life years is .6 years for the 75-year-old woman, compared with 1.7 years for the 55-year-old man. An online interactive calculator for individualized predictions and treatment benefits is available here.

## Discussion

The LIFE-Preserved model for the prediction of short-term and lifetime risks of HF hospitalization or CV death was derived and externally validated using data from 48 394 patients with HFpEF. The LIFE-Preserved model allows the estimation of HF hospitalization-free survival and life expectancy (*Structured Graphical Abstract*). The model can be combined with the best available evidence from clinical trials to predict individualized benefit from guideline-recommended therapies expressed as HF hospitalization-free life years gained. An interactive calculator of the LIFE-Preserved model has been provided until future implementation of the model in online calculators, such as the CE-marked medical device U-prevent.

Of the datasets used, SwedeHF was considered the most representative cohort for routine clinical practice, providing real-world incidences of HF hospitalizations and CV and non-CV death. Therefore, the model without any recalibration factors (i.e. based on the event rates in the SwedeHF registry) is recommended for use in daily clinical practice. Predicted risks of HF hospitalization or CV death from the LIFE-Preserved model were in line with the observed incidence in VA, TOPCAT-Americas, and HF-Particles. In EMPEROR-Preserved and the NHSE SDE, recalibration of risks was required to reflect observed incidence in those cohorts. This was likely due to the stricter inclusion criteria in clinical trials, differences in patient inclusion and baseline measurement during the COVID period, and/or geographical differences across data sources. Importantly, should new and more broadly representative HFpEF registries become available in the future, the model should be readily recalibrated to reflect local risk levels and increasing use of novel HF therapies. Such recalibration can be easily incorporated into the online implementation, allowing the LIFE-Preserved model to remain accurate and adaptable as new data sources emerge.

Regarding clinical implementation, the LIFE-Preserved model is specifically designed to support clinical decision-making by identifying patients at highest risk of a next or first HF hospitalization or CV death. It enables clinicians to estimate prognosis and to guide timely initiation of evidence-based therapies. Importantly, as patient risk profiles evolve over time and after hospitalizations, the model’s iterative use allows for updated prognostic assessments and supports ongoing decisions about therapy adjustments, rather than serving as a one-time risk estimate. Consequently, the model can also be applied to patients who have already experienced a HF hospitalization, using their updated clinical profile. The model can be used easily through the online risk calculator and will also be incorporated into the CE-marked medical device U-prevent platform, further facilitating its accessibility across diverse clinical settings.

The LIFE-Preserved model has several strengths compared with existing risk models for the HFpEF population. First, LIFE-Preserved can predict risk at any time horizon as well as lifetime risk. Prior risk models are limited to predicting outcomes over a 1- or 2-year period,^15,19^ whereas HFpEF is a chronic disease and therapies are usually continued lifelong. The limited prediction horizon of previously available risk models does not cover the full-time horizon relevant for clinical decision-making. LIFE-Preserved showed good discriminative ability in external validation, which was shown in the current study for up to 10 years in external validation data. Pooled C-statistics were higher in trial data than in registry data, likely reflecting more precise phenotyping of HFpEF patients in trials, rather than true differences in model performance. In addition, the higher observed incidence in the registries could also have contributed to a lower C-statistics in registries.^45^ In comparison to the existing PREDICT-HFpEF model, the LIFE-Preserved model showed a better discrimination (see Supplementary data online, *Table S10*). LIFE-Preserved also demonstrated adequate calibration in both registry and trial data, making it a useful tool for guiding long-term treatment decisions in this population.

Second, LIFE-Preserved can estimate lifetime treatment benefits. As a clinical example, the estimated treatment benefit for SGLT2 inhibitors is presented, based on applying clinical trial HRs to modelled absolute risks rather than observed treatment effects in the derivation cohort, where usage was very low. These estimates provide a framework for understanding potential lifetime treatment benefit. The lifetime treatment benefit, defined as the gain in HF hospitalization-free life years, may provide a more intuitive measure for individuals considering preventive treatment. It has been shown that treatment benefits can contribute to risk communication and reduce decisional conflict for individuals.^46^ This information improves shared decision-making, enabling patients and physicians to collaboratively balance expected treatment benefits against practical considerations, such as medication burden, potential side effects, and financial costs.

Third, the LIFE-Preserved model derives strength from its derivation in SwedeHF, which is one of the largest datasets of HFpEF patients with long-term follow-up for HF hospitalizations and deaths. SwedeHF includes patients from the real-world setting, including both patients from the outpatient setting and patients who are hospitalized at inclusion. The LIFE-Preserved model demonstrated good calibration in both groups, indicating that it can be reliably used for stable HF patients as well as for those recently hospitalized. The high observed event rates for HF hospitalization or CV death as well as for non-CV death in SwedeHF are consistent with what has been reported in other real-world HFpEF populations.^3^ This ensures high generalizability and relevance to real-world patient populations. Risk factor measurements reflect clinical care, which has been shown to improve model performance and transferability.^47^ By comparison, trial data usually have more complete baseline data but may be less representative of patients as seen in clinical practice, which could lead to potential misalignment of predicted and observed risk. This is likely due to strict exclusion criteria in trials, which may introduce selection by excluding less healthy individuals. For example, the LIFE-Preserved model, developed in real-world data, overestimated the risk of non-CV death in those trials where both patients with severe comorbidities and limited life expectancy were excluded (TOPCAT-Americas and EMPEROR-Preserved).

Fourth, LIFE-Preserved is externally validated in multiple data sources, both trials and observational cohorts, spanning different European and non-European risk regions. Its consistent performance across these settings supports its applicability and reliability in a wide range of healthcare systems and patient populations.

Lastly, the model’s development and validation are in line with the PROBAST guideline^48^ and TRIPOD statement.^42^ It follows established statistical practices, mirroring those used in the LIFE-HF model for HFrEF.^26^ This results in two complementary models that can predict both short-term and lifetime risks for two major HF subtypes, providing a more holistic tool for clinical decision-making. We recognize that HF with mildly reduced ejection fraction (HFmrEF) was not included in the LIFE-Preserved model, as clinical characteristics and underlying mechanisms more closely resemble HFrEF and predictor–risk relationships may therefore differ from HFpEF.^49,50^ Future studies should evaluate the performance of HFrEF models (such as LIFE-HF) in HFmrEF to complete the continuum of risk prediction for HF patients. Clinical predictors were predefined and selected based on an extensive literature review and a multidisciplinary expert panel, consisting of cardiologists, primary care physicians, epidemiologists, and health service researchers. Selected predictors are readily available in clinical practice, which enhances the model’s practical implementation across all lines of care. Furthermore, LIFE-Preserved adjusts for the competing risk of non-CV death. In older and high-risk populations, such as those with HF, not accounting for competing risks has been shown to lead to an overestimation of predicted risks, which could result in overtreatment.^34^ By accounting for competing risks, the resulting risk estimates better reflect actual patient outcomes. Lastly, sex-specific derivation of coefficients and baseline hazards ensures that LIFE-Preserved captures differences in relative effects of certain predictors and in patterns of risk progression between men and women. External validation showed that model performance was consistent across sexes, confirming that LIFE-Preserved is appropriately calibrated for both men and women in clinical practice.

Potential limitations also need to be considered. First, registries such as SwedeHF can be selective and have imperfect coverage. SwedeHF captured 31% of prevalent HF diagnoses in 2020.^27,51^ Although this may introduce some degree of selection, the registry nevertheless reflects a broad and clinically relevant HF population. Importantly, the strong and consistent performance of our model in the external validation cohorts provides reassurance that any potential limitations in representativeness did not materially affect model development or applicability. Second, due to the use of routinely collected data, there was a relevant proportion of missing data for several model predictor variables, such as NT-proBNP, BMI, and NYHA class. However, missing data were imputed using recommended multiple imputation methods, and the model was derived by pooling the coefficients across all 45 imputed datasets. The model performance in external validation supports the robustness of these derived coefficients. Third, although the median follow-up of 1.8 years may appear short, this primarily reflects the rapid occurrence of events rather than inadequate observation time. The large population provided substantial long-term data, with follow-up exceeding 4.2 years in 25% (*N* = 5083) of patients (see Supplementary data online, *Figure S13A*), ensuring sufficient overlapping data across age groups for reliable lifetime risk estimation. Consequently, survival curves remained stable well beyond 10 years (see Supplementary data online, *Figure S13B*). Although true lifetime risk estimates cannot be validated, because no contemporary cohort has sufficiently long follow-up, the model demonstrated good calibration up to 10 years of follow-up in external validation, and the methodology used has been shown to yield accurate risks up to at least 17 years.^35^ Fourth, in the current examples, individualized treatment benefits were shown only for the outcome of HF hospitalization or CV death, given its relevance and modifiability in HFpEF. Recommendations for these therapies are presently based on observed reductions in HF hospitalizations and CV mortality rather than total mortality. In addition, individualized treatment benefits were estimated using a fixed treatment effect (i.e. a single treatment effect regardless of patient characteristics). This approach assumes no heterogeneity in relative treatment response among HFpEF patients, as trials and meta-analyses to date have not reported major differences. However, when applying the model in clinical practice to calculate treatment effects, it should be used alongside the most up-to-date evidence, including any potential heterogeneity in treatment response, should such evidence emerge. The LIFE-Preserved model is designed to provide a framework to be used alongside the latest evidence on therapy effectiveness in this group. The online implementation will be continuously updated, integrating new treatments and updating HRs when new evidence emerges.

In conclusion, the LIFE-Preserved model can predict risk at any time horizon as well as lifetime risk of HF hospitalization or CV death and HF hospitalization-free life expectancy for individual patients with HFpEF. Combining the LIFE-Preserved model with treatment effects from trials or meta-analyses allows for individualized estimates of treatment benefit. Thus, this model could serve as a tool to improve the management of patients with HFpEF by facilitating personalized medicine and shared decision-making.

## Supplementary Material

ehag182_Supplementary_Data

## Appendix

### ESC Cardiovascular Risk Collaboration

Prof. Emanuele Angelantonio (Department of Public Health and Primary Care, University of Cambridge, Victor Phillip Dahdaleh Heart & Lung Research Institute), Prof. Ana Abreu (Hospital de Santa Maria, Faculty of Medicine, Lisbon), Prof. Frank Visseren (Department of Vascular Medicine, University Medical Center Utrecht), Prof. Maryam Kavousi (Department of Vascular Medicine, University Medical Center Utrecht), Prof. John William McEvoy (Preventive Cardiology, School of Medicine, University of Galway), Prof. Angela Wood (Department of Public Health and Primary Care, University of Cambridge, Victor Phillip Dahdaleh Heart & Lung Research Institute).

## Declarations

### Data Availability

Data used for the current study are available upon reasonable request and approval of the individual data sources or collaborative groups (availability and methods may depend on the specific data source used).

The accompanying R code and a minimal reproducible example using synthetic data are available upon reasonable request to the corresponding author.

The North East—Newcastle and North Tyneside 2 research ethics committee provided ethical approval for the CVD-COVID-UK/COVID-IMPACT research programme (REC No. 20/NE/0161) to access, within secure trusted research environments, unconsented, whole-population, anonymized data from electronic health records collected as part of patients’ routine healthcare. The data used in this study are available in NHS England’s Secure Data Environment (SDE) service for England, but as restrictions apply, they are not publicly available (https://digital.nhs.uk/services/secure-data-environment-service). The CVD-COVIDUK/COVID-IMPACT research programme, led by the BHF Data Science Centre (https://bhfdatasciencecentre.org/), received approval to access data in NHS England’s SDE service for England from the Advisory Group for Data (AGD) (https://digital.nhs.uk/about-nhs-digital/corporateinformation-and-documents/advisory-group-for-data)—formerly the Independent Group Advising on the Release of Data (IGARD)—via an application made in the Data Access Request Service (DARS) Online system (reference number: DARS-NIC-381078-Y9C5K) (https://digital.nhs.uk/services/data-access-request-servicedars/dars-products-and-services). The CVD-COVID-UK/COVID-IMPACT Approvals & Oversight Board (https://bhfdatasciencecentre.org/areas/cvd-covid-uk-covid-impact/) subsequently granted approval to this project (CCU004) to access the data within NHS England’s SDE service for England. The anonymized data used in this study were made available to accredited researchers only. Those wishing to gain access to the data should follow the application process of the relevant national data custodian. The analysis using data for England was performed according to a pre-specified analysis plan published on GitHub, along with the phenotyping code lists, and data curation and analysis code (https://github.com/BHFDSC/CCU004_05).
