# Supplementary material for: Risk prediction in patients with heart failure with preserved ejection fraction: the LIFE-Preserved model
Source: Eur Heart J. 2026 Mar 11;47(34):4773–88. doi: 10.1093/eurheartj/ehag182 (PMC13549728; doi:10.1093/eurheartj/ehag182)
Supplement: Supplementary file 1 [file ehag182_supplementary_data.zip › REVISED2_Reitsma_LIFE-Preserved_Supplements.docx]

**SUPPLEMENTARY MATERIAL**

Supplement to: Risk prediction in patients with heart failure with preserved ejection fraction: the LIFE-Preserved model

Table of contents

[Supplementary Methods	2](#_Toc223601247)

[*External validation cohorts*	2](#_Toc223601248)

[*Predictor selection*	3](#_Toc223601249)

[*Missing data*	4](#_Toc223601250)

[*Model derivation*	5](#_Toc223601251)

[*Lifetime prediction using lifetable method*	5](#_Toc223601252)

[*Individualized treatment benefit*	7](#_Toc223601253)

[*Recalibration*	7](#_Toc223601254)

[*Calibration plots*	8](#_Toc223601255)

[Supplementary Tables	9](#_Toc223601256)

[*Table S1. Data sources and diagnosis codes for predictor definitions in SwedeHF	9*](#_Toc223601257)

[*Table S2. Outcome definitions in the study populations	10*](#_Toc223601258)

[*Table S3. Predictors of the LIFE-Preserved model	12*](#_Toc223601259)

[*Table S4. Missingness of predictor variables in SwedeHF registry at time of inclusion	13*](#_Toc223601260)

[*Table S5. Suggested hazard ratios for estimation of benefit from recommended therapies	14*](#_Toc223601261)

[*Table S6. Differences between in- and out-patient inclusions in SwedeHF	15*](#_Toc223601262)

[*Table S7a. Age-specific baseline survival for HF hospitalization or CV death	16*](#_Toc223601263)

[*Table S7b. Age-specific baseline survival for non-CV death	17*](#_Toc223601264)

[*Table S8. Linear predictors of the LIFE-Preserved model	18*](#_Toc223601265)

[*Table S9. Discrimination of the LIFE-Preserved model for both outcomes in subgroups	20*](#_Toc223601266)

[*Table S10. C-statistics of LIFE-Preserved compared to PREDICT-HFpEF in EMPEROR-Preserved	21*](#_Toc223601267)

[*Table S11. EO ratios in external validation prior to recalibration	22*](#_Toc223601268)

[Supplementary Figures	23](#_Toc223601269)

[*Figure S1. Flowchart of study population in SwedeHF Registry	23*](#_Toc223601270)

[*Figure S2. Review of predictors in earlier models	24*](#_Toc223601271)

[*Figure S3. Effect of additional predictors on model discrimination for HF hospitalization or CV death	25*](#_Toc223601272)

[*Figure S4. Functional form of transformed predictors and age interactions for primary outcome	26*](#_Toc223601273)

[*Figure S5. Smoothing of baseline survival	28*](#_Toc223601274)

[*Figure S6. Internal calibration in SwedeHF	29*](#_Toc223601275)

[*Figure S7. C-statistics for HF hospitalization or CV death in sex-specific subgroups	32*](#_Toc223601276)

[*Figure S8. External calibration of HF hospitalization or CV death in women	33*](#_Toc223601277)

[*Figure S9. External calibration of HF hospitalization or CV death in men	34*](#_Toc223601278)

[*Figure S10. External calibration of non-CV death	35*](#_Toc223601279)

[*Figure S11. External calibration of HF hospitalization or CV death in subgroups	37*](#_Toc223601280)

[*Figure S12. External calibration of LIFE-Preserved during COVID-19 period in the NHSE SDE	38*](#_Toc223601281)

[*Figure S13. Descriptives for follow-up time for HF hospitalization or CV death in SwedeHF	39*](#_Toc223601282)

[Supplementary References	40](#_Toc223601283)

# Supplementary Methods

## *External validation cohorts*

The EMPEROR-Preserved trial (Empagliflozin Outcome Trial in Patients with Chronic Heart Failure with Preserved Ejection Fraction)^1^ is a randomized, double-blind, placebo-controlled trial investigating the effect of empagliflozin, a SGLT2-inhibitor, in patients with HFpEF or HFmrEF. The trial included 5,988 patients with symptomatic heart failure and a LVEF greater than 45% between 2017 and 2020, across 23 countries worldwide. Empagliflozin significantly reduced the primary outcome of HF hospitalization or CV death compared to placebo. For the current study we included all HFpEF patients (LVEF ≥50% at baseline) aged 40-89 years (n = 3,955).

The TOPCAT trial (Treatment of Preserved Cardiac Function Heart Failure with an Aldosterone Antagonist)^2^ is a randomized, double-blind, placebo-controlled trial investigating the effect of spironolactone in patients with HFpEF or HFmrEF. The trial included 3,445 patients with symptomatic heart failure and a LVEF of 45% or higher between 2006 and 2012. The trial included patients from around the world, including North and South America, Russia, and Repuplic of Georgia. In the main analyses spironolactone did not significantly reduce the primary outcome of HF hospitalization or CV death. Post-hoc analyses of the trial revealed lower event rates in patients included in Russia or Georgia, raising concerns about the validity of HF diagnosis in those regions.^3^ Therefore, in the current study, only those patients from the United-States, Canada, Argentina, and Brazil (TOPCAT-Americas) were included for external validation. We included all HFpEF patients (LVEF ≥50% at baseline) aged 40-89 years from those regions (n = 1,553).

The HF-Particles cohort in Poland is a sub cohort of the larger EP-PARTICLES study, a comprehensive, population-based epidemiological project conducted in Eastern Poland.^4^ The project focuses on assessing the impact of environmental risk factors, such as air pollution, noise, light exposure, socio-economic conditions, and climate change, on the incidence, progression, and mortality of cardiovascular diseases (CVD). The HF-PARTICLES cohort includes patients with HFpEF residing in the Podlaskie Voivodeship, a region characterized by a high proportion of elderly residents and prolonged exposure to airborne pollutants, commonly referred to as “Polish smog.” At the same time, the region has one of the highest life expectancies in the country, adding complexity to the health risk analysis. Patients were enrolled in this prospective observational study between 2012 and 2016. The follow-up period lasted until January 15, 2023. The follow-up strategy was mixed, including both telephone interviews and event validation using official records from the Central Statistical Office in Olsztyn and the National Health Fund in Białystok.

A cohort of US Veterans (n = 27,443) that underwent elective isolated coronary artery bypass grafting at 41 VA medical centers nationally between January 2010 – December 2019 was identified with a follow-up till March 2020 (pre-Covid in the US).^5^ From these patients, using a combination of left ventricular ejection fraction, use of diuretic therapy, ICD codes, and NYHA class, 4642 with pre-existing HFpEF at the time of surgery were identified. The primary endpoint was a combination of the first event of heart failure hospitalization or cardiovascular mortality with a competing endpoint of non-cardiovascular mortality. Non-fatal events were identified using the ICD9, ICD10 codes; only the primary cause for admission was considered to identify cause-specific admissions. The Department of Veterans Affairs updates the vital status of US Veterans that are enrolled in their healthcare program; this information is accurate as it is implemented to determine continuation of healthcare benefits. The VA also directly collects information regarding death notifications from the Center for Medicare and Medicaid Services, Social Security Administration, and the National Death Index. The vital status data was linked with approval to the National Death Repository, a VA supported database that provides cause of death information from death certificates. The ICD9, ICD10 codes in the first three columns were considered to define whether patients died due to cardiovascular causes.

The NHS England Secure Data Environment (NHSE SDE) data resource includes linked electronic health records from primary care (General Practice data for Pandemic Planning and Research, GDPPR), secondary care (Hospital Episode Statistics Admitted Patient Care), Tertiary care (Audits from the National Institute for Cardiovascular Outcomes Research (NICOR) National Cardiac Audit Programme, including the National Heart Failure Audit) and deaths (Office of National Statistics death registrations) covering >96% of the English population and 98% of all general practices. Data were accessed via the British Heart Foundation Data Science Centre’s [CVD-COVID-UK/COVID-IMPACT Consortium](https://eur05.safelinks.protection.outlook.com/?url=https%3A%2F%2Fbhfdatasciencecentre.org%2Fareas%2Fcvd-covid-uk-covid-impact%2F&data=05%7C02%7CT.H.Reitsma-3%40umcutrecht.nl%7C9ea41f0a550a4eeb5d2408de396522e3%7Cdcdf4a3dd0c04a6394cf781981249be5%7C0%7C0%7C639011303765425364%7CUnknown%7CTWFpbGZsb3d8eyJFbXB0eU1hcGkiOnRydWUsIlYiOiIwLjAuMDAwMCIsIlAiOiJXaW4zMiIsIkFOIjoiTWFpbCIsIldUIjoyfQ%3D%3D%7C0%7C%7C%7C&sdata=zmZqzLIDiHuUvr5BEy7RQbQ%2BffTzhJOog0N0DRVqZF8%3D&reserved=0) and is available for COVID-related research questions. Our objectives were to assess the performance of the LIFE-Preserved risk model for overall and heart failure hospitalisation-free survival in patients with Heart failure with preserved ejected fraction (HFpEF) during the pandemic period (2020-2022) and the post-pandemic period (2022-2024). In particular we hypothesised that the LIFE-Preserved risk model may underestimate risk of heart failure hospitalisation and heart failure death endpoints during the pandemic period due to the direct and indirect impacts of COVID-19 on cardiovascular disease and vulnerable heart failure patients, but likely to perform better in the post-pandemic period. Two retrospective cohorts of HFpEF patients aged 40-90 were created, the first (n=1,870) spanning the COVID-19 period, from January 1^st^2020 to March 1^st^ 2022, and the second (n=17,005) a post COVID cohort from March 2^nd^ 2022 to December 2024. Heart failure patients were defined as those hospitalised with heart failure as the primary diagnosis in any consultant episode of an in-patient admission to a National Health Service (NHS) hospital.  Patients were classified with HFpEF using left ventricular ejection fraction of >50%. Further details can be found in the supplementary material of a previously published article.^6^ Baseline measurements were selected as those being the closest to the start of the cohort and measured between 1-year prior up to 1-year after start of the cohort. This analysis was performed according to a pre-specified analysis plan with the code lists to define variables and analysis code published on github:  [https://github.com/BHFDSC/CCU004_05.](https://eur05.safelinks.protection.outlook.com/?url=https%3A%2F%2Fgithub.com%2FBHFDSC%2FCCU004_05&data=05%7C02%7CT.H.Reitsma-3%40umcutrecht.nl%7C9ea41f0a550a4eeb5d2408de396522e3%7Cdcdf4a3dd0c04a6394cf781981249be5%7C0%7C0%7C639011303765469047%7CUnknown%7CTWFpbGZsb3d8eyJFbXB0eU1hcGkiOnRydWUsIlYiOiIwLjAuMDAwMCIsIlAiOiJXaW4zMiIsIkFOIjoiTWFpbCIsIldUIjoyfQ%3D%3D%7C0%7C%7C%7C&sdata=gDYBxHTTFWmrgrY0rA2RR0zPDZfmgmfIkJOl6TBLc5s%3D&reserved=0) The data used in this validation sub-study are available in NHS England’s Secure Data Environment (SDE) service for England, but as restrictions apply they are not publicly available.^7^ The CVD-COVID-UK/COVID-IMPACT research programme, led by the BHF Data Science Centre^8^, received approval to access data in NHS England’s SDE service for England from the Advisory Group for Data (AGD) – formerly the Independent Group Advising on the Release of Data (IGARD) – via an application made in the Data Access Request Service (DARS) Online system (ref. DARS-NIC-381078-Y9C5K). ^9^ The CVD-COVID-UK/COVID-IMPACT Approvals & Oversight Board^10^ subsequently granted approval to this project (CCU004_05) to access the data within NHS England’s SDE service for England. The anonymised data used in this study were made available to accredited researchers only. Those wishing to gain access to the data should follow the application process of the relevant national data custodian. The North East - Newcastle and North Tyneside 2 research ethics committee provided ethical approval for the CVD-COVID-UK/COVID-IMPACT research programme (REC No 20/NE/0161) to access, within secure trusted research environments, unconsented, whole-population, anonymised data from electronic health records collected as part of patients’ routine healthcare.

## *Predictor selection*

An overview on existing prediction models targeting HFpEF patients was made prior to analyses. Literature was searched and six existing prediction models were identified. These models included the PREDICT-HFpEF model^11^, the EMPEROR-Preserved model^12^, the 3A3B score^13^, the MAGGIC score^14^, the I-Preserve model^15^ and the CHARM model^16^. All models included patients with HFpEF in the derivation cohorts and predicted clinically relevant outcomes. Of these models, all candidate and final predictors were retrieved and ordered based on the frequency of their occurrences (Figure S2). All predictors that were included two or more times as final predictor in different models were regarded as core predictors to be included in LIFE-Preserved (Table S3). An expert panel, consisting of cardiologists, primary care physicians, epidemiologists, and health services researchers, evaluated the core predictors based on recent literature, clinical practice experience, and perceived importance for longer-term predictions. The expert panel recommended adding five additional predictors: atrial fibrillation, cerebrovascular disease (previous stroke/TIA), loop diuretic use, systolic blood pressure, and duration of heart failure (Table S3). To assess the added predictive value of each of the five additional predictors an extended model was derived including all the core predictors plus the additional predictor. The discriminative performance of the core model and the five extended models was assessed by calculating Harrell’s C-statistics in the SwedeHF Registry. Additional predictors that showed a statistically significant increase in C-statistics, with a significance level of 0.05, were included as core predictors for the final model.

## *Missing data*

Missing data in the SwedeHF Registry is reported in Table S3. As the proportion of missing data in the derivation data was high, this was handled using multivariate imputation by chained equations (MICE) with 45 iterations and 45 imputations. Two separate imputation models were created, one for women (MICE.1) and one for men (MICE.2), to maintain congeniality with the sex-specific model derivation. The primary outcome of HF hospitalization or CV death and the competing outcome of non-CV death were both included using a Nelson-Aalan estimator.^17^ There was no missingness in outcome data. Transformations of continuous predictors were included in the imputation model and imputed passively. Body mass index was passively imputed as well based on the imputed values of weight and height. Convergence of the imputation model was assessed visually through inspecting of trace plots and showed stable parameter estimations across the 45 iterations. The imputation model only included variables that were used as predictors and not variables listed in the baseline table only. Continuous predictors were imputed using predictive mean matching, binary variables using logistic regression, and multi-level variables using polytomous regression. The need for non-linear transformations was checked across all 45 multiple imputed datasets and the best transformation was chosen based on the AIC. For each variable, the transformation that was most frequently identified as optimal across the 45 imputed datasets was used in the final models. Cox proportional hazards models were applied to the 45 imputed datasets and pooled using Rubin’s rules.^18^ Baseline hazards were estimated using the pooled coefficients in one of the 45 imputed datasets as there were no missing in baseline age, follow-up time and events status in SwedeHF.

In external validation, missing data was handled by single imputation in EMPEROR-Preserved, HF-Particles and VA as proportions of missing were low. In HF-Particles NT-proBNP was not available, and the sex-specific median of the outpatients in the derivation cohort was used as this was an outpatient cohort and the outpatients are more likely reflecting true values for the NT-proBNP in the outpatient setting (1400 pg/mL for women and 1202 pg/mL for men). In VA no information about NT-proBNP and heartrate was available, and the sex-specific median of the derivation cohort was used (NT-proBNP 2120 pg/mL for women and 1755 pg/mL for men; heartrate 72 bpm for women and 70 bpm for men). In TOPCAT-Americas and the NHSE SDE missing data was handled by multiple imputation, because missingness was high. For all imputation models in external validation, all predictors were included as well as a Nelson-Aalan estimator.^17^ For validation in multiple imputed data, model performance parameters were also pooled using Rubin’s rules. Pooled EO-ratios were used for model recalibration.

## *Model derivation*

The need for non-linear transformations for continuous predictors was investigated by comparing Akaike’s information criterium (AIC) of the model without transformations to models with, respectively, squared, or cubic polynomials, and logarithmic, or square root transformations. Schoenfeld residuals were assessed visually to assess the proportionality of the hazard-ratios. Interactions between age for specific predictors were added in case of violations of proportionality. The need for transformations and age-interactions was assessed on the population as a whole (i.e. women and men together) before proceeding with sex specific development. The estimation of the coefficients was performed separately for both sexes.

Sex-specific baseline survival probabilities were obtained for both outcomes by estimating 3-month interval survival over the complete age range of the derivation population (40-90 years). All baseline survival probabilities were estimated for a reference patient with all binary predictors at 0 and the following levels for continuous predictors: age at baseline 40 years, NT-proBNP 1250 pg/mL, BMI 25 kg/m^2^, heartrate 80 bpm, eGFR 65 mL/min/1.73m^2^, haemoglobin 8 mmol/L. The same reference values were used for both men and women. The baseline survival probabilities were then smoothed using a non-linear function and weighted by the number of patients in each age-interval. For the outcome of HF hospitalization or CV death, a Locally Estimated Scatterplot Smoothing (LOESS) function was used to smoothen the baseline survival probabilities. For the competing outcome of non-CV death, an exponential (Nonlinear Least Squares) function was used to smoothen the baseline survival probabilities to ensure survival probabilities remained below one, as a survival probability of 100% is not clinically possible.

## *Lifetime prediction using lifetable method*

The lifetable method combines the baseline hazards with the coefficients from the cause-specific Cox models to calculate competing risk adjusted individual risk for HF hospitalization or CV death and non-CV death. These are the steps for lifetime prediction using the lifetable method, with an example of a lifetable below.

1. Using individual risk factor levels the linear predictors for both outcomes (LP_HFH+CV death_ and LP_non-CV death_) can be estimated using the algorithms provided in Table S8.
2. By combining the linear predictors with the age-specific 3-month survival probabilities, provided in Table S7, from the patient’s current age up to the maximum age in the cohort (90 years), the interval specific survival probabilities for both outcomes for an individual patient can be calculated.

For HF hospitalization or CV death (column 2 in the example):

S_HFH+CV death_ (t) = (S_0, HFH+CV death_ (t)^exp(LP_HFH+CV death_)

For non-CV death (column 3 in the example):

S_non-CV death_ (t) = (S_0,non-CV death_ (t)^exp(LP_non-CV death_)

In these functions, t represents a remaining 3-month interval for the individual patient until age 90. S0 (t) represents the baseline survival probability for the specific outcome and that specific interval t. The “^” denotes exponentiation, whereby the baseline survival probability (S0 (t)) is raised to the power of the individual relative hazard (exp(LP)).

3. Next, interval-specific event-free survival probability can be calculated for each 3-month interval (column 4):

S_interval_(t) = 1 – (1 - S_HFH+CV death_) – (1 - S_non-CV death_)

4. Cumulative event-free survival probabilities can be calculated by multiplying the interval-specific survival probabilities of all previous intervals (column 5). By definition, the cumulative event-free survival started at 100% at the current age of a person in the life-table. Cumulative event-free survival was calculated until the maximum age of 90 years. HF hospitalization-free life expectancy was defined as the age where the estimated cumulative survival drops below 50%, which was 80.4 years in the example lifetable.
5. The competing risk adjustment for both outcomes was done by first estimating the probability of any event in each interval from the event-free survival function. This probability was then split into two outcomes: HF hospitalization or CV death, and non-CV death. The split was based on the relative risk of each outcome in that interval.
6. Cumulative risks can then be calculated by adding the adjusted interval-specific risks of all previous intervals (columns 7 and 8).

| **Age** | **Interval specific survival of HFH or CV death (%)** | **Interval specific survival of non-CV death (%)** | **Interval specific event-free survival (%)** | **Cumulative event-free survival (%)** | **Interval specific risk of HFH or CV death (%)** | **Interval specific risk non-CV death (%)** | **Cumulative risk of HF hospitalization or CV death (%)** | **Cumulative risk of non-CV death (%)** |
| --- | --- | --- | --- | --- | --- | --- | --- | --- |
| 75 | 97.44 | 99.44 | 96.89 | 100 | 2.56 | 0.56 | 0 | 0 |
| 75.25 | 97.45 | 99.43 | 96.88 | 96.89 | 2.47 | 0.55 | 2.56 | 0.56 |
| 75.5 | 97.46 | 99.42 | 96.88 | 93.86 | 2.39 | 0.54 | 5.03 | 1.11 |
| 75.75 | 97.46 | 99.41 | 96.87 | 90.93 | 2.31 | 0.54 | 7.41 | 1.65 |
| 76 | 97.47 | 99.40 | 96.87 | 88.09 | 2.23 | 0.53 | 9.72 | 2.19 |
| 76.25 | 97.48 | 99.39 | 96.87 | 85.33 | 2.15 | 0.52 | 11.95 | 2.72 |
| 76.5 | 97.48 | 99.38 | 96.86 | 82.66 | 2.08 | 0.51 | 14.1 | 3.24 |
| 76.75 | 97.49 | 99.37 | 96.85 | 80.06 | 2.01 | 0.51 | 16.18 | 3.75 |
| 77 | 97.49 | 99.36 | 96.85 | 77.54 | 1.94 | 0.5 | 18.19 | 4.26 |
| …... |  |  |  |  |  |  |  |  |
| 88 | 97.44 | 98.56 | 96.00 | 16.40 | 0.42 | 0.24 | 63.25 | 20.35 |
| 88.25 | 97.43 | 98.53 | 95.97 | 15.74 | 0.4 | 0.23 | 63.67 | 20.59 |
| 88.5 | 97.43 | 98.51 | 95.93 | 15.11 | 0.39 | 0.23 | 64.08 | 20.82 |
| 88.75 | 97.42 | 98.48 | 95.90 | 14.49 | 0.37 | 0.22 | 64.46 | 21.04 |
| 89 | 97.41 | 98.45 | 95.86 | 13.90 | 0.36 | 0.22 | 64.84 | 21.26 |
| 89.25 | 97.40 | 98.42 | 95.82 | 13.32 | 0.35 | 0.21 | 65.2 | 21.48 |
| 89.5 | 97.39 | 98.39 | 95.78 | 12.77 | 0.33 | 0.21 | 65.54 | 21.69 |
| 89.75 | 97.38 | 98.36 | 95.75 | 12.23 | 0.32 | 0.20 | 65.88 | 21.89 |
| 90 |  |  |  | 11.71 |  |  | 66.2 | 22.09 |

Risk factor profile for this lifetable: a 75-year-old woman with atrial fibrillation, BMI 30 kg/m², haemoglobin 7.8 mmol/L, eGFR 55 mL/min/1.73 m², heart rate 75 bpm, and NT-proBNP 2120 pg/mL. She had a prior hospitalization for heart failure but none in the past 6 months, with no ischemic heart disease, COPD, or diabetes, was a non-smoker, and had NYHA class II symptoms.

These steps can be filled in for a specific patient, for example the woman from Figure 3 in the main text. Columns 2 and 3 represent the interval-specific survival probabilities for HF hospitalization or CV death, and for non-CV death, respectively, derived in Step 2. These are combined to obtain the interval-specific event-free survival probability (column 4). The cumulative event-free survival probability (column 5) is then calculated by multiplying the survival probabilities of all previous intervals. Columns 6 and 7 show the interval-specific risks for HF hospitalization or CV death and non-CV death, respectively, while columns 8 and 9 present the corresponding competing risk-adjusted cumulative risks. In this example, starting from age 75 years, the cumulative probability of surviving without any event until age 77 is 77.54%. Two-year risks (until age 77) are 18.2% for HF hospitalization or CV death and 4.3% for non-CV death. Lifetime risk (risk until age 90) are 66.2% for HF hospitalization or CV death and 22.1% for non-CV death. In this example lifetime risk would be the same as 15-year risks, but for a 50-year old patient lifetime risk would represent 40-year risk.

## *Individualized treatment benefit*

It has been previously shown that risk estimations can be combined with relative treatment effects from trials to estimate absolute individualized treatment effects.^19–22^

First, the patient’s risk for HF hospitalization or CV death and non-CV death should be calculated for the current risk profile. Causal (relative) hazard ratios (HR) can be obtained for both outcomes from trial or meta-analyses. The HRs can be combined with the lifetable estimation by introducing this after Step 1.

LP_with treatment_ = LP_current treatment_ + ln(HR)

In this paper the treatment benefit for SGLT-2 inhibitors (HR 0.81) were calculated, but other treatment options can be calculated as well (see Table S5 for suggested HRs for other therapies). Estimating the effect of more than one therapy, the HR’s for both therapies should be multiplied and are assumed independent. In this paper, treatments were assumed to have no effect on non-CV death.

The individualized absolute risk reduction (ARR) for a new treatment was then calculated as the difference between the predicted risk on current treatment and the new treatment regimen, which can be done for all prediction horizons (e.g., 2-year risks). Similarly, individualized lifetime benefit was calculated as the difference between the predicted HF hospitalization-free life expectancy on current and new treatment.

*Integrating treatment effects for patients on current treatment*

If a patient is already receiving one or more evidence-based treatments, the corresponding treatment effects can be incorporated into the LIFE-Preserved model in the same way. By applying the corresponding hazard ratios for the current treatment to the predicted risks, the risk estimates reflect the patient’s current treatment regimen. This approach was also applied to patients in the active-treatment arm of the EMPEROR-Preserved trial, where the SGLT2 inhibitor treatment effect (HR 0.79)^1^ was integrated into the predicted risks.

## *Recalibration*

The LIFE-Preserved model can be recalibrated to other data sources if its estimates are deemed not representative (e.g. other geographical locations or specialized care). Recalibration requires the sex-specific expected-observed (EO) ratio’s for both outcomes. The process involves the following steps:

1. Recalibration can be performed for different time horizons. The horizon must be determined in advance and should align with the intended clinical use of the model.

2. Using the LIFE-Preserved model, estimate the predicted risk of HF hospitalization or CV death and non-CV death at the selected time horizon. Then, calculate the mean predicted risk for men and women separately among patients with an available prediction at that horizon. Note: The LIFE-Preserved model provides predictions up to age 90. For example, only patients aged 88 years or younger have a valid 2-year predicted risk. Therefore, the mean predicted risk should be calculated only among patients with available predictions at the selected horizon.

3. Estimate the observed risk for men and women separately for the same patients who have a predicted risk at the selected time horizon. This can be done by using the *cuminc* function in R.

4. Calculate the sex-specific EO ratio’s by dividing the sex-specific mean predicted risk (Step 2) by the sex-specific observe risk (Step 3).

5. Recalibrate the model by subtracting the natural logarithm of the sex-specific EO ratio from the linear predictor for both outcomes (LP – ln(EO)).

## *Calibration plots*

Calibration plots were made accounting for competing risks. First, predicted risks were ordered from low to high. Risks were rounded to no decimals (e.g., 38.1% was rounded to 38%) and all unique predicted risks in the data were extracted. At each of the unique risk estimates the number of observed events was estimated using the *cuminc* function R, obtaining both the observed incidence at that particular risk estimate as well as the standard error for the estimation. Following sequencing along all predicted risks a loess regression was performed using the number of observations at each unique risk as weights. Similarly, the confidence intervals were obtained by using the standard errors to obtain 95% confidence intervals, to which the same loess regression was applied to obtain smoothed estimates. As some unique risks have few observations a dynamic window was used that included the observations from the unique risk estimate and those within ±1% from that unique risk. This window was expanded dynamically (by multiplying the window of 1% with 1.5 etc.) until it contained at least two primary events and two other outcomes (either competing events or censored cases). For visualization purposes, the predicted risks were truncated at the 99th percentile to avoid unstable estimates in regions with very few individuals. The background histogram in the plots shows the distribution of predicted risks in the population, allowing readers to assess the density of predictions across different risk levels.

# Supplementary Tables

## Table S1. Data sources and diagnosis codes for predictor definitions in SwedeHF

| Variable | Data source |
| --- | --- |
| Index year | SwedeHF |
| Sex | SwedeHF |
| Age | SwedeHF |
| Duration of HF | SwedeHF |
| Previous HF hospitalizaton | NPR: ICD-10 I110, I130, I132, I255, I420, I423, I425-9, I43, I50, J81, K761, R570 ^b^ |
| LVEF | SwedeHF |
| NYHA class | SwedeHF |
| BMI | SwedeHF |
| Systolic blood pressure | SwedeHF |
| Heart rate | SwedeHF |
| Creatinine | SwedeHF |
| Hemoglobin | SwedeHF |
| NT-proBNP | SwedeHF |
| Smoking status | SwedeHF |
| Diabetes | SwedeHF |
| Ischemic heart disease | SwedeHF; NPR: ICD-9/10 410-4, I20-5, Z951, Z955 Procedure FNG, FNA, FNB, FNC, FND, FNE, FNF, FNH ^a^ |
| Atrial fibrillation/flutter | SwedeHF; NPR: ICD-10 I48 ^a^ |
| Cerebrovascular disease (stroke or TIA) | NPR: ICD-9/10 430-4, 438, I60-4, I690-4, G45 ^a^ |
| COPD | NPR: ICD-10 J40-4 ^a^ |

Abbreviations: HF = heart failure; NPR = national patient register; NYHA = New York Heart Association; LVEF = Left ventricular ejection fraction; COPD = Chronic obstructive pulmonary disease; BMI = Body mass index; NT-proBNP = N-terminal pro-B-type natriuretic peptide, TIA = transient ischemic attack.

^a^ Derived from diagnoses/operations in all positions, in-patient and out-patient specialty care within 5 years prior to index (ICD-10)/1987 (ICD-9) unless stated otherwise.
^b^ Hospitalizations are derived from diagnoses in main position, in-patient care.

## Table S2. Outcome definitions in the study populations

| Study | Outcome evaluation & definitions |
| --- | --- |
| SwedeHF Registry | *Evaluation*: Vital status, date of death and cause of death is extracted from the Cause of Death Register.^23^ Data on HF hospitalizations are extracted by linking with the National Patient Register.^24^  *Hospitalization for HF:* First registered hospitalization with ICD-10 code I110, I130, I132, I255, I420, I423, I425, I426, I427, I428, I429, I43, I50, J81, K761, or R57 in primary position.  *CV death*: Registered death with ICD-10 code I00-99, J81, K761, R57, or G45 as cause of death. |
| Emperor-Preserved | *Evaluation:* Outcomes were adjudicated by a blinded clinical endpoints committee.  *Hospitalization for HF:* First admission to hospital with a minimum stay of 12 hours with new or worsening HF presentation (indicated by one or more symptoms^a^ AND at least two physical examination findings^b^ OR one physical examination finding and at least one laboratory criterion^c^) requiring treatment^d^.  *CV death:* Death due to acute MI, or sudden cardiac death, worsening HF (i.e. pump failure), stroke, complications of a cardiovascular procedure, cardiovascular haemorrhage, death due to other cardiovascular causes (e.g. pulmonary embolism or peripheral arterial disease). |
| TOPCAT | *Evaluation:* Outcomes were adjudicated by a blinded clinical endpoints committee.  *First hospitalization for HF*: First unexpected overnight stay to an acute care facility with exacerbation of HF (indicated by at least one symptom^e^ AND at least 2 signs^f^) requiring treatment^g^.  *CV death:* Death due to fatal MI, pump failure, sudden death, presumed CV death (death occurring when patient was last seen >24 hours before death and presumed CV death), stroke, pulmonary embolism, CV-procedure related, death due to other cardiovascular causes. |
| HF-Particles | *Evaluation:* HF hosptializations and cause of death were extracted from the Central Statistical Office in Olsztyn and the National Health Fund in Białystok.  *Hospitalization for HF:* First registered hospitalization with ICD-10 code I110, I130, I132, I255, I420, I423, I425, I426, I427, I428, I429, I43, I50, J81, K761, or R57.  *CV death*: Registered death with ICD-10 code I00-99, J81, K761, R57, or G45. |
| VA | *Evaluation:* HF hosptializations were identified by admissions to VA medical centers or admissions to non-VA hospitals when the charges were supported by the VA. Cause and dates of death were extracted from the National Death Registry, updated directly by the Department of VA and receiving information from the Center for Medicare and Medicaid Services, Social Security Administration, and the National Death Index.  *Hospitalization for HF:* First registered hospitalization with ICD-10 code I110, I130, I132, I255, I420, I423, I425, I426, I427, I428, I429, I43, I50, J81, K761, or R57 in primary position.  *CV death*: Registered death with ICD-10 code I00-99, J81, K761, R57, or G45 |
| NHSE SDE | *Evaluation:* HF hosptializations and cause of death were extracted by linking electronic health records from secondary care (Hospital Episode Statistics Admitted Patient Care), Tertiary care (Audits from the National Institute for Cardiovascular Outcomes Research (NICOR) National Cardiac Audit Programme) and deaths (Office of National Statistics death registrations).  *Hospitalization for HF:* First registered hospitalization with ICD-10 code I110, I130, I132, I255, I420, I423, I425, I426, I427, I428, I429, I43, I50, J81, K761, or R57 in primary position.  *CV death*: Registered death with ICD-10 code I00-99, J81, K761, R57, or G45 |

Abbreviations: HF = heart failure; CV = cardiovascular; MI = myocardial infarction.

^a^ Symptoms: dyspnoea, decreased exercise tolerance, fatigue, other symptoms of worsened end-organ perfusion (e.g. dizziness, volume overload).

^b^ Physical examination findings: peripheral oedema, increasing abdominal distention or ascites, pulmonary rales/crackles/crepitations, increased jugular venous pressure and/or hepatojugular reflux, S3 gallop, clinically significant or rapid weight gain thought to be related to fluid retention.

^c^ Laboratory evidence: increased B-type natriuretic peptide (BNP) or N-terminal pro-BNP (NT-proBNP) concentrations.

^d^ Treatment: augmentation in oral diuretic therapy, intravenous diuretic or vasoactive agent, mechanical or surgical intervention (circulatory support, mechanical fluid removal).

^e^ Symptoms: increasing dyspnoea on exertion, worsening orthopnoea, paroxysmal nocturnal dyspnoea, increasing fatigue/worsening exercise tolerance, altered mental status or other (specify).

^f^ Signs: peripheral oedema, elevated jugular venous pressure, radiologic signs of HF, increasing abdominal distension or ascites, pulmonary oedema or rales, rapid weight gain, hepatojugular reflux, S3 gallop, elevated BNP or NT-proBNP, other (specify).

^g^ Treatment with either intravenous diuretics, intravenous vasodilators, intravenous inotropes, mechanical fluid removal or insertion of an intra-aortic balloon pump for hemodynamic compromise.

## Table S3. Predictors of the LIFE-Preserved model

a. Core predictors (n=13)

Core predictors were selected based on a literature overview of existing prediction models (supplementary Methods).

| Demographic | Clinical features of HF | Medical history | Physical signs | Laboratory tests |
| --- | --- | --- | --- | --- |
| Age | NYHA class | Diabetes mellitus | BMI | NT-proBNP |
| Sex |  | COPD | Heartrate | Haemoglobin |
|  |  | Prior HF hospitalization |  | Estimated GFR |
|  |  | Smoking status |  |  |
|  |  | Ischemic heart disease |  |  |

b. Additional predictors (n=5)

Additional predictors were identified after review by an expert panel (supplementary Methods).

| Demographic | Clinical features of HF | Medical history | Physical signs | Laboratory tests |
| --- | --- | --- | --- | --- |
|  | Duration of HF | Atrial fibrillation | Systolic blood pressure |  |
|  | Use of loop diuretics | Cerebrovascular disease |  |  |

c. Final predictors (n=14)

Atrial fibrillation showed a statistically significant increase in C-statistics for the outcome of HF hospitalization or CV death compared to the core model and was therefore included in the final model (Figure S3).

| Demographic | Clinical features of HF | Medical history | Physical signs | Laboratory tests |
| --- | --- | --- | --- | --- |
| Age | NYHA class | Diabetes mellitus | BMI | NT-proBNP |
| Sex |  | COPD | Heartrate | Haemoglobin |
|  |  | Prior HF hospitalization |  | Estimated GFR |
|  |  | Smoking status |  |  |
|  |  | Ischemic heart disease |  |  |
|  |  | Atrial fibrillation |  |  |

Abbreviations: NYHA: New York Heart Association; COPD = Chronic obstructive pulmonary disease; HF: heart failure; BMI: Body mass index; NT-proBNP: N-terminal pro-B-type natriuretic peptide; eGFR: estimated glomerular filtration rate.

## Table S4. Missingness of predictor variables in SwedeHF registry at time of inclusion

|  | Missing (%) |
| --- | --- |
| Age | 0% |
| Duration of HF | 0% |
| Sex | 0% |
| Current smoking | 29% |
| Body mass index (kg/m2)^a^ | 36% |
| Height (cm) | 33% |
| Weight (kg) | 11% |
| Systolic blood pressure (mmHg) | 2.4% |
| Heart rate (bpm) | 5.6% |
| NYHA class | 39% |
| Prior HF hospitalization ^*^ | 0% |
| Diabetes mellitus | 0% |
| Ischemic heart disease ^*^ | 0% |
| COPD ^*^ | 0% |
| Atrial fibrillation ^*^ | 0% |
| Cerebrovascular disease ^*^ | 0% |
| NT-proBNP (pg/mL) | 45% |
| Haemoglobin (mmol/L) | 4.6% |
| Creatinine (µmol/L)^b^ | 2.6% |
| Loop diuretics | 34% |

* For comorbidities and previous hospitalization, which was retrieved by linking with National Patient Register in Sweden, the absence of an in-patient or out-patient specialty care diagnosis was assumed to indicate no history of the respective condition.

Abbreviations: HF = heart failure; NYHA = New York Heart Association; COPD = chronic obstructive pulmonary disease; NT-proBNP = N-terminal pro-B-type natriuretic peptide.

^a^ BMI was passively imputed by imputing height and weight and calculating BMI afterwards.

^b^ Missingness of creatinine is displayed as this was used to calculate estimated glomerular filtration rate (calculated with the CKD-EPI formula 2009).

## Table S5. Suggested hazard ratios for estimation of benefit from recommended therapies

| Treatment | Trial(s)/meta-analysis | Hazard ratio for HF hospitalization or CV death | Hazard ratio for non-CV death | Applies to: |
| --- | --- | --- | --- | --- |
| SGLT2 inhibitor | Meta-analysis^25^, including:  - DELIVER trial^26^  - EMPEROR-Preserved trial^1^ | 0.81 | Assumed no effect | All HFpEF patients |
| Finerenone | FINEARTS-HF trial^27^ | 0.84 | Assumed no effect | All HFpEF patients |
| Semaglutide | Pooled analysis of the SELECT, FLOW, STEP-HFpEF, and STEP-HFpEF DM randomised trials^28^ | 0.69 | Assumed no effect | HFpEF patients with BMI higher than 27 |
| Tirzepatide | SUMMIT-trial^29^ | 0.62 | Assumed no effect | HFpEF patients with BMI higher than 27 |

Abbreviations: SGLT2 = sodium/glucose cotransporter 2.

Note that all trials used in this population had additional in- and exclusion criteria. The suggested effect sizes may therefore not be applicable to the complete population. Please check the latest evidence of the respective trials before applying these for individual patients. In addition, some of the above drugs may not (yet) be generally recommended by current guidelines.

## Table S6. Differences between in- and out-patient inclusions in SwedeHF

|  | **Out-patient** | **In-patient** |
| --- | --- | --- |
|  | n = 9808 | n = 10524 |
| Age (years) | 75 ± 10 | 78 ± 9 |
| Women | 4,660 (48%) | 5,821 (55%) |
| Current smokers | 778 (7.9%) | 979 (9.3%) |
| Physical signs |  |  |
| Body mass index (kg/m2) | 28 ± 6 | 28 ± 6 |
| Systolic blood pressure (mmHg) | 132 ± 20 | 133 ± 22 |
| Diastolic blood pressure (mmHg) | 74 ± 12 | 73 ± 12 |
| Heart rate (bpm) | 71 ± 14 | 76 ± 16 |
| Clinical features of HF |  |  |
| NYHA class |  |  |
| I | 1,497 (15%) | 1,578 (15%) |
| II | 4,852 (50%) | 4,441 (42%) |
| III | 3,458 (35%) | 4,505 (43%) |
| IV | 133 (1.4%) | 578 (5.5%) |
| Duration of HF (years) | 0.3 [0.0-2.5] | 0.1 [0.0-2.9] |
| Left bundle branch block | 647 (6.6%) | 705 (6.7%) |
| Medical history |  |  |
| Prior HF hospitalization | 3,608 (37%) | 7,613 (72%) |
| Recent HF hospitalization (≤ 6 months) | 2,022 (21%) | 7,009 (67%)**^*^** |
| Diabetes mellitus | 2,321 (24%) | 3,420 (32%) |
| Ischemic heart disease | 4,085 (42%) | 5,275 (50%) |
| COPD | 1,237 (13%) | 2,074 (20%) |
| Obesity | 3,286 (34%) | 3,239 (31%) |
| Atrial fibrillation | 6,056 (62%) | 7,044 (67%) |
| Cerebrovascular disease | 1,461 (15%) | 2,223 (21%) |
| Peripheral artery disease | 787 (8.0%) | 1,117 (11%) |
| Valve disease | 3,045 (31%) | 3,753 (36%) |
| Dilated cardiomyopathy | 545 (5.6%) | 181 (1.7%) |
| Laboratory measurements |  |  |
| NT-proBNP (pg/mL) | 1,298 [529-2,672] | 2,760 [1,340-5,459] |
| Haemoglobin (mmol/L) | 8.2 ± 1.0 | 7.7 ± 1.1 |
| Estimated GFR (mL/min/1.73m2) | 52 [39 - 67] | 48 [34 - 64] |
| Medication use |  |  |
| Loop diuretics | 6,932 (71%) | 9,199 (87%) |
| ACE-inhibitor / ARB | 7,838 (80%) | 6,930 (66%) |
| Beta-blocker | 8,086 (82%) | 8,394 (80%) |
| Statin | 4,453 (45%) | 4,336 (41%) |
| MRA | 2,980 (30%) | 3,121 (30%) |
| Digoxin | 1153 (12%) | 1,711 (16%) |
| Nitrate | 1,104 (11%) | 1,853 (18%) |
| Device therapy | 287 (2.9%) | 113 (1.1%) |
| ARNI | 62 (0.6%) | 6 (0.1%) |
| SGLT2 inhibitor | 70 (0.7%) | 23 (0.2%) |
| GLP1 receptor agonist | 65 (0.7%) | 58 (0.6%) |
| Follow-up time (years) | 2.1 [0.8-4.7] | 1.4 [0.3-3.8] |
| Incidence of HFH/CV death | 3,090 (32%) | 6,251 (59%) |
| Event rate of HFH/CV death per 1000 PY | 98 | 229 |
| Incidence of non-CV death | 2,013 (21%) | 4,028 (38%) |
| Event rate of non-CV death per 1000 PY | 55 | 108 |
| * In-patients can be admitted to the hospital for different reasons than HF, which is why this number is not 100%.  Data are presented as number (%), mean ±standard deviation, median [interquartile range] as appropriate. Abbreviations: HF = heart failure; bpm = beats per minute; NYHA = New York Heart Association; COPD = chronic obstructive pulmonary disease; NT-proBNP = N-terminal pro-B-type natriuretic peptide; GFR = glomerular filtration rate (calculated with CKD-EPI formula 2009); ACE = angiotensin-converting enzyme; ARB = angiotensin receptor blocker; ARNI = angiotensin receptor-neprilysin inhibitor; MRA = mineralocorticoid receptor antagonist; SGLT2 = sodium-glucose cotransporter 2; GLP1 = glucagon-like peptide 1; HFH = heart failure hospitalization; CV = cardiovascular; PY = person years. a Obesity is defined as BMI ≥30 kg/m2. b Cerebrovascular disease consist of prior stroke or prior transient ischemic attack (TIA). c Device therapy includes cardiac resynchronization therapy and implantable cardioverter-defibrillator. d Event rate of hospitalization for heart failure or cardiovascular death per 1000 person-years. | | |

## Table S7a. Age-specific baseline survival for HF hospitalization or CV death

| Age (years) | 3-month survival for women | 3-month survival for men | Age | 3-month survival for women | 3-month survival for men | Age | 3-month survival for women | 3-month survival for men |
| --- | --- | --- | --- | --- | --- | --- | --- | --- |
| 40 | 0,991152 | 0,993511 | 56,75 | 0,996568 | 0,996148 | 73,5 | 0,997555 | 0,997026 |
| 40,25 | 0,991332 | 0,993579 | 57 | 0,99657 | 0,996162 | 73,75 | 0,997564 | 0,997036 |
| 40,5 | 0,991513 | 0,993646 | 57,25 | 0,996573 | 0,996176 | 74 | 0,997572 | 0,997045 |
| 40,75 | 0,991694 | 0,993714 | 57,5 | 0,996576 | 0,996189 | 74,25 | 0,99758 | 0,997053 |
| 41 | 0,991873 | 0,99378 | 57,75 | 0,99658 | 0,996203 | 74,5 | 0,997587 | 0,997061 |
| 41,25 | 0,99205 | 0,993846 | 58 | 0,996585 | 0,996216 | 74,75 | 0,997595 | 0,997069 |
| 41,5 | 0,992225 | 0,993912 | 58,25 | 0,996591 | 0,99623 | 75 | 0,997602 | 0,997077 |
| 41,75 | 0,992396 | 0,993976 | 58,5 | 0,996598 | 0,996243 | 75,25 | 0,997609 | 0,997085 |
| 42 | 0,992563 | 0,994039 | 58,75 | 0,996609 | 0,996257 | 75,5 | 0,997616 | 0,997093 |
| 42,25 | 0,992724 | 0,994101 | 59 | 0,996623 | 0,99627 | 75,75 | 0,997622 | 0,997101 |
| 42,5 | 0,99288 | 0,994162 | 59,25 | 0,99664 | 0,996283 | 76 | 0,997628 | 0,997108 |
| 42,75 | 0,993028 | 0,994221 | 59,5 | 0,996659 | 0,996296 | 76,25 | 0,997634 | 0,997115 |
| 43 | 0,993169 | 0,994279 | 59,75 | 0,996681 | 0,996309 | 76,5 | 0,997639 | 0,997121 |
| 43,25 | 0,993305 | 0,994335 | 60 | 0,996704 | 0,996321 | 76,75 | 0,997645 | 0,997127 |
| 43,5 | 0,993441 | 0,994391 | 60,25 | 0,996727 | 0,996334 | 77 | 0,997649 | 0,997132 |
| 43,75 | 0,993576 | 0,994446 | 60,5 | 0,996751 | 0,996346 | 77,25 | 0,997654 | 0,997136 |
| 44 | 0,993708 | 0,9945 | 60,75 | 0,996775 | 0,996359 | 77,5 | 0,997658 | 0,99714 |
| 44,25 | 0,993839 | 0,994554 | 61 | 0,996798 | 0,996371 | 77,75 | 0,997662 | 0,997143 |
| 44,5 | 0,993968 | 0,994607 | 61,25 | 0,996819 | 0,996383 | 78 | 0,997666 | 0,997146 |
| 44,75 | 0,994093 | 0,994659 | 61,5 | 0,996839 | 0,996396 | 78,25 | 0,997669 | 0,997149 |
| 45 | 0,994215 | 0,99471 | 61,75 | 0,996856 | 0,996408 | 78,5 | 0,997672 | 0,997152 |
| 45,25 | 0,994334 | 0,994761 | 62 | 0,996873 | 0,99642 | 78,75 | 0,997675 | 0,997154 |
| 45,5 | 0,994448 | 0,99481 | 62,25 | 0,996891 | 0,996432 | 79 | 0,997677 | 0,997156 |
| 45,75 | 0,994558 | 0,994858 | 62,5 | 0,996911 | 0,996444 | 79,25 | 0,99768 | 0,997157 |
| 46 | 0,994662 | 0,994906 | 62,75 | 0,996931 | 0,996456 | 79,5 | 0,997682 | 0,997158 |
| 46,25 | 0,994763 | 0,994952 | 63 | 0,996952 | 0,996467 | 79,75 | 0,997683 | 0,997159 |
| 46,5 | 0,994862 | 0,994998 | 63,25 | 0,996973 | 0,996479 | 80 | 0,997685 | 0,997159 |
| 46,75 | 0,99496 | 0,995043 | 63,5 | 0,996994 | 0,996491 | 80,25 | 0,997686 | 0,997159 |
| 47 | 0,995054 | 0,995087 | 63,75 | 0,997015 | 0,996502 | 80,5 | 0,997687 | 0,997159 |
| 47,25 | 0,995147 | 0,99513 | 64 | 0,997036 | 0,996514 | 80,75 | 0,997688 | 0,997158 |
| 47,5 | 0,995237 | 0,995172 | 64,25 | 0,997056 | 0,996526 | 81 | 0,997689 | 0,997157 |
| 47,75 | 0,995324 | 0,995214 | 64,5 | 0,997075 | 0,996538 | 81,25 | 0,997689 | 0,997155 |
| 48 | 0,995409 | 0,995254 | 64,75 | 0,997093 | 0,996551 | 81,5 | 0,997689 | 0,997153 |
| 48,25 | 0,995491 | 0,995294 | 65 | 0,99711 | 0,996564 | 81,75 | 0,997689 | 0,997151 |
| 48,5 | 0,995569 | 0,995334 | 65,25 | 0,997126 | 0,996578 | 82 | 0,997689 | 0,997149 |
| 48,75 | 0,995645 | 0,995372 | 65,5 | 0,997142 | 0,996592 | 82,25 | 0,997688 | 0,997146 |
| 49 | 0,995717 | 0,99541 | 65,75 | 0,997158 | 0,996608 | 82,5 | 0,997687 | 0,997143 |
| 49,25 | 0,995786 | 0,995448 | 66 | 0,997173 | 0,996623 | 82,75 | 0,997686 | 0,997139 |
| 49,5 | 0,995851 | 0,995484 | 66,25 | 0,997188 | 0,996639 | 83 | 0,997685 | 0,997135 |
| 49,75 | 0,995914 | 0,99552 | 66,5 | 0,997203 | 0,996655 | 83,25 | 0,997683 | 0,99713 |
| 50 | 0,995973 | 0,995555 | 66,75 | 0,997218 | 0,996671 | 83,5 | 0,997681 | 0,997126 |
| 50,25 | 0,99603 | 0,995589 | 67 | 0,997232 | 0,996687 | 83,75 | 0,997679 | 0,99712 |
| 50,5 | 0,996084 | 0,995622 | 67,25 | 0,997246 | 0,996702 | 84 | 0,997676 | 0,997115 |
| 50,75 | 0,996135 | 0,995655 | 67,5 | 0,99726 | 0,996717 | 84,25 | 0,997673 | 0,997109 |
| 51 | 0,996184 | 0,995686 | 67,75 | 0,997274 | 0,996731 | 84,5 | 0,99767 | 0,997102 |
| 51,25 | 0,99623 | 0,995717 | 68 | 0,997288 | 0,996744 | 84,75 | 0,997667 | 0,997095 |
| 51,5 | 0,996274 | 0,995747 | 68,25 | 0,997302 | 0,996758 | 85 | 0,997664 | 0,997088 |
| 51,75 | 0,996315 | 0,995777 | 68,5 | 0,997316 | 0,996772 | 85,25 | 0,99766 | 0,997081 |
| 52 | 0,996354 | 0,995805 | 68,75 | 0,99733 | 0,996786 | 85,5 | 0,997656 | 0,997073 |
| 52,25 | 0,996391 | 0,995834 | 69 | 0,997344 | 0,996801 | 85,75 | 0,997651 | 0,997065 |
| 52,5 | 0,996423 | 0,99586 | 69,25 | 0,997359 | 0,996816 | 86 | 0,997647 | 0,997056 |
| 52,75 | 0,996449 | 0,995884 | 69,5 | 0,997373 | 0,996831 | 86,25 | 0,997642 | 0,997047 |
| 53 | 0,99647 | 0,995907 | 69,75 | 0,997387 | 0,996846 | 86,5 | 0,997637 | 0,997037 |
| 53,25 | 0,996486 | 0,995927 | 70 | 0,997401 | 0,996861 | 86,75 | 0,997631 | 0,997028 |
| 53,5 | 0,996497 | 0,995946 | 70,25 | 0,997414 | 0,996875 | 87 | 0,997626 | 0,997017 |
| 53,75 | 0,996506 | 0,995963 | 70,5 | 0,997428 | 0,996889 | 87,25 | 0,99762 | 0,997007 |
| 54 | 0,996512 | 0,99598 | 70,75 | 0,99744 | 0,996902 | 87,5 | 0,997614 | 0,996996 |
| 54,25 | 0,996517 | 0,995996 | 71 | 0,997452 | 0,996914 | 87,75 | 0,997607 | 0,996985 |
| 54,5 | 0,996521 | 0,996011 | 71,25 | 0,997464 | 0,996925 | 88 | 0,9976 | 0,996973 |
| 54,75 | 0,996524 | 0,996026 | 71,5 | 0,997476 | 0,996937 | 88,25 | 0,997593 | 0,996961 |
| 55 | 0,996528 | 0,996041 | 71,75 | 0,997487 | 0,996949 | 88,5 | 0,997586 | 0,996948 |
| 55,25 | 0,996534 | 0,996057 | 72 | 0,997497 | 0,99696 | 88,75 | 0,997579 | 0,996935 |
| 55,5 | 0,996541 | 0,996073 | 72,25 | 0,997508 | 0,996972 | 89 | 0,997571 | 0,996922 |
| 55,75 | 0,996549 | 0,996089 | 72,5 | 0,997518 | 0,996984 | 89,25 | 0,997563 | 0,996908 |
| 56 | 0,996556 | 0,996104 | 72,75 | 0,997528 | 0,996995 | 89,5 | 0,997554 | 0,996894 |
| 56,25 | 0,996561 | 0,996119 | 73 | 0,997537 | 0,997006 | 89,75 | 0,997546 | 0,99688 |
| 56,5 | 0,996564 | 0,996134 | 73,25 | 0,997546 | 0,997016 |  |  |  |

| Age | 3-month survival for women | 3-month survival for men | Age | 3-month survival for women | 3-month survival for men | Age | 3-month survival for women | 3-month survival for men |
| --- | --- | --- | --- | --- | --- | --- | --- | --- |
| 40 | 0,998331 | 0,998386 | 56,75 | 0,994428 | 0,993318 | 73,5 | 0,981397 | 0,972337 |
| 40,25 | 0,998301 | 0,998351 | 57 | 0,994327 | 0,993174 | 73,75 | 0,981059 | 0,971744 |
| 40,5 | 0,99827 | 0,998316 | 57,25 | 0,994224 | 0,993028 | 74 | 0,980715 | 0,971139 |
| 40,75 | 0,998238 | 0,99828 | 57,5 | 0,994119 | 0,992879 | 74,25 | 0,980365 | 0,97052 |
| 41 | 0,998206 | 0,998243 | 57,75 | 0,994012 | 0,992726 | 74,5 | 0,980008 | 0,969889 |
| 41,25 | 0,998174 | 0,998205 | 58 | 0,993903 | 0,99257 | 74,75 | 0,979645 | 0,969243 |
| 41,5 | 0,998141 | 0,998167 | 58,25 | 0,993793 | 0,992411 | 75 | 0,979276 | 0,968584 |
| 41,75 | 0,998107 | 0,998127 | 58,5 | 0,99368 | 0,992248 | 75,25 | 0,978899 | 0,967911 |
| 42 | 0,998073 | 0,998087 | 58,75 | 0,993565 | 0,992082 | 75,5 | 0,978516 | 0,967223 |
| 42,25 | 0,998038 | 0,998046 | 59 | 0,993448 | 0,991913 | 75,75 | 0,978126 | 0,966521 |
| 42,5 | 0,998002 | 0,998004 | 59,25 | 0,993329 | 0,991739 | 76 | 0,977729 | 0,965804 |
| 42,75 | 0,997966 | 0,997962 | 59,5 | 0,993208 | 0,991562 | 76,25 | 0,977325 | 0,965071 |
| 43 | 0,997929 | 0,997918 | 59,75 | 0,993085 | 0,991381 | 76,5 | 0,976913 | 0,964322 |
| 43,25 | 0,997891 | 0,997873 | 60 | 0,992959 | 0,991197 | 76,75 | 0,976494 | 0,963558 |
| 43,5 | 0,997853 | 0,997828 | 60,25 | 0,992832 | 0,991008 | 77 | 0,976067 | 0,962777 |
| 43,75 | 0,997814 | 0,997781 | 60,5 | 0,992701 | 0,990815 | 77,25 | 0,975632 | 0,961979 |
| 44 | 0,997774 | 0,997734 | 60,75 | 0,992569 | 0,990619 | 77,5 | 0,97519 | 0,961164 |
| 44,25 | 0,997734 | 0,997685 | 61 | 0,992434 | 0,990417 | 77,75 | 0,97474 | 0,960332 |
| 44,5 | 0,997693 | 0,997636 | 61,25 | 0,992297 | 0,990212 | 78 | 0,974281 | 0,959482 |
| 44,75 | 0,997651 | 0,997585 | 61,5 | 0,992157 | 0,990002 | 78,25 | 0,973814 | 0,958614 |
| 45 | 0,997608 | 0,997533 | 61,75 | 0,992014 | 0,989788 | 78,5 | 0,973339 | 0,957727 |
| 45,25 | 0,997565 | 0,99748 | 62 | 0,991869 | 0,989569 | 78,75 | 0,972854 | 0,956821 |
| 45,5 | 0,99752 | 0,997426 | 62,25 | 0,991722 | 0,989346 | 79 | 0,972362 | 0,955896 |
| 45,75 | 0,997475 | 0,997371 | 62,5 | 0,991571 | 0,989117 | 79,25 | 0,97186 | 0,954951 |
| 46 | 0,99743 | 0,997315 | 62,75 | 0,991418 | 0,988884 | 79,5 | 0,971349 | 0,953985 |
| 46,25 | 0,997383 | 0,997257 | 63 | 0,991263 | 0,988646 | 79,75 | 0,970829 | 0,952999 |
| 46,5 | 0,997335 | 0,997198 | 63,25 | 0,991104 | 0,988403 | 80 | 0,970299 | 0,951992 |
| 46,75 | 0,997287 | 0,997138 | 63,5 | 0,990942 | 0,988154 | 80,25 | 0,96976 | 0,950963 |
| 47 | 0,997238 | 0,997077 | 63,75 | 0,990778 | 0,9879 | 80,5 | 0,969211 | 0,949912 |
| 47,25 | 0,997188 | 0,997014 | 64 | 0,990611 | 0,987641 | 80,75 | 0,968652 | 0,948839 |
| 47,5 | 0,997137 | 0,996951 | 64,25 | 0,99044 | 0,987376 | 81 | 0,968082 | 0,947743 |
| 47,75 | 0,997085 | 0,996885 | 64,5 | 0,990266 | 0,987106 | 81,25 | 0,967503 | 0,946623 |
| 48 | 0,997032 | 0,996818 | 64,75 | 0,99009 | 0,986829 | 81,5 | 0,966913 | 0,945479 |
| 48,25 | 0,996978 | 0,99675 | 65 | 0,98991 | 0,986547 | 81,75 | 0,966312 | 0,94431 |
| 48,5 | 0,996923 | 0,996681 | 65,25 | 0,989727 | 0,986259 | 82 | 0,965701 | 0,943117 |
| 48,75 | 0,996867 | 0,996609 | 65,5 | 0,98954 | 0,985964 | 82,25 | 0,965078 | 0,941898 |
| 49 | 0,99681 | 0,996537 | 65,75 | 0,98935 | 0,985664 | 82,5 | 0,964444 | 0,940653 |
| 49,25 | 0,996752 | 0,996463 | 66 | 0,989157 | 0,985356 | 82,75 | 0,963798 | 0,939381 |
| 49,5 | 0,996693 | 0,996387 | 66,25 | 0,98896 | 0,985043 | 83 | 0,963141 | 0,938082 |
| 49,75 | 0,996633 | 0,996309 | 66,5 | 0,988759 | 0,984722 | 83,25 | 0,962472 | 0,936755 |
| 50 | 0,996572 | 0,99623 | 66,75 | 0,988555 | 0,984395 | 83,5 | 0,96179 | 0,9354 |
| 50,25 | 0,99651 | 0,996149 | 67 | 0,988348 | 0,98406 | 83,75 | 0,961096 | 0,934016 |
| 50,5 | 0,996446 | 0,996067 | 67,25 | 0,988136 | 0,983719 | 84 | 0,96039 | 0,932602 |
| 50,75 | 0,996382 | 0,995983 | 67,5 | 0,987921 | 0,98337 | 84,25 | 0,959671 | 0,931157 |
| 51 | 0,996316 | 0,995897 | 67,75 | 0,987701 | 0,983013 | 84,5 | 0,958939 | 0,929682 |
| 51,25 | 0,996249 | 0,995809 | 68 | 0,987478 | 0,982649 | 84,75 | 0,958193 | 0,928175 |
| 51,5 | 0,996181 | 0,995719 | 68,25 | 0,987251 | 0,982278 | 85 | 0,957434 | 0,926636 |
| 51,75 | 0,996112 | 0,995627 | 68,5 | 0,987019 | 0,981898 | 85,25 | 0,956661 | 0,925064 |
| 52 | 0,996041 | 0,995533 | 68,75 | 0,986783 | 0,98151 | 85,5 | 0,955874 | 0,923458 |
| 52,25 | 0,995969 | 0,995438 | 69 | 0,986543 | 0,981114 | 85,75 | 0,955073 | 0,921818 |
| 52,5 | 0,995896 | 0,99534 | 69,25 | 0,986299 | 0,980709 | 86 | 0,954258 | 0,920142 |
| 52,75 | 0,995822 | 0,99524 | 69,5 | 0,98605 | 0,980296 | 86,25 | 0,953427 | 0,918431 |
| 53 | 0,995746 | 0,995138 | 69,75 | 0,985797 | 0,979873 | 86,5 | 0,952581 | 0,916683 |
| 53,25 | 0,995669 | 0,995034 | 70 | 0,985539 | 0,979442 | 86,75 | 0,95172 | 0,914898 |
| 53,5 | 0,99559 | 0,994927 | 70,25 | 0,985277 | 0,979001 | 87 | 0,950844 | 0,913074 |
| 53,75 | 0,99551 | 0,994819 | 70,5 | 0,985009 | 0,978551 | 87,25 | 0,949951 | 0,911211 |
| 54 | 0,995428 | 0,994708 | 70,75 | 0,984737 | 0,978092 | 87,5 | 0,949043 | 0,909309 |
| 54,25 | 0,995345 | 0,994594 | 71 | 0,98446 | 0,977622 | 87,75 | 0,948117 | 0,907365 |
| 54,5 | 0,995261 | 0,994478 | 71,25 | 0,984178 | 0,977143 | 88 | 0,947175 | 0,90538 |
| 54,75 | 0,995175 | 0,99436 | 71,5 | 0,983891 | 0,976653 | 88,25 | 0,946216 | 0,903352 |
| 55 | 0,995087 | 0,994239 | 71,75 | 0,983598 | 0,976153 | 88,5 | 0,94524 | 0,901281 |
| 55,25 | 0,994998 | 0,994116 | 72 | 0,9833 | 0,975642 | 88,75 | 0,944246 | 0,899166 |
| 55,5 | 0,994907 | 0,99399 | 72,25 | 0,982997 | 0,97512 | 89 | 0,943233 | 0,897005 |
| 55,75 | 0,994815 | 0,993861 | 72,5 | 0,982688 | 0,974587 | 89,25 | 0,942203 | 0,894798 |
| 56 | 0,994721 | 0,993729 | 72,75 | 0,982374 | 0,974042 | 89,5 | 0,941153 | 0,892543 |
| 56,25 | 0,994625 | 0,993595 | 73 | 0,982054 | 0,973486 | 89,75 | 0,940085 | 0,890241 |
| 56,5 | 0,994527 | 0,993458 | 73,25 | 0,981728 | 0,972917 |  |  |  |

## Table S7b. Age-specific baseline survival for non-CV death

## Table S8. Linear predictors of the LIFE-Preserved model

a. Women

| **Linear predictor (LP) for HF hospitalization or CV death**  LP_HFH+CV death_ = | | |
| --- | --- | --- |
|  | | 0.72502722807 (if first-year adjustment^a^) + 0.04772439010*(age_baseline – 40) + 1.64364704252 (if diabetes mellitus) + 0.14672305231 (if current smoker) + 0.23991524942*(ln(NT-proBNP) – ln(1250)) - 0.22020535287*(BMI – 25) + 0.00642547860*(BMI^2 – 25^2) - 0.00005586338*(BMI^3 – 25^3) + 0.00852461199*(heartrate – 80) - 0.00004003875*(heartrate^2 – 80^2) - 0.01209077516*(eGFR – 65) + 0.00004952883*(eGFR^2 – 65^2) - 0.62508756234*(Hb – 8) + 0.03637319680*(Hb^2 – 8^2) + 0.21895731862 (if NYHA class III or IV) + 0.26588488439 (if COPD) + 0.31854162088 (if previously hospitalized for HF) + 0.17906151258 (if ischemic heart disease) + 0.18726322701 (if atrial fibrillation) - 0.01744246329*age_vector (if diabetes mellitus) - ln(1.014965) + ln(cause-specific hazard ratio of risk-modifying therapy)^b^ |
| **Linear predictor (LP) for non-CV death**  LP_non-CV death_ = | | |
|  | | 0.2322771358 (if first-year adjustment^a^) - 0.0308672516*(age_baseline – 40) + 0.2999288430 (if diabetes mellitus) - 0.0395281553 (if current smoker) + 0.2278187543*(ln(NT-proBNP) – ln(1250)) - 0.4714866682*(BMI – 25) + 0.0128711280*(BMI^2 – 25^2) - 0.0001148799*(BMI^3 – 25^3) + 0.0253361734*(heartrate – 80) - 0.0001102053*(heartrate^2 – 80^2) - 0.0279838659*(eGFR – 65) + 0.0001939482*(eGFR^2 – 65^2) - 2.0944659127*(Hb –8) + 0.1269888188*(Hb^2 – 8^2) + 0.3170897239 (if NYHA class III or IV) + 0.7196992131 (if COPD) + 0.1343886560 (if previously hospitalized for HF) - 0.1464743725 (if ischemic heart disease) - 0.1559688553 (if atrial fibrillation) - ln(1.266831) |
|  | Age_baseline in years at moment of prediction; age_vector is a sequence of ages (in rounded 3-month intervals) from the patient’s current age up to 89.75 years; NT-proBNP in pg/mL; BMI in kg/m^2^; heartrate in bpm; eGFR: estimated glomerular filtration rate in mL/min/1.73 m^2^; Hb (haemoglobin) in mmol/L.  ^a^ Add a first-year adjustment for the first four time intervals (3 months * 4 = 1 year) for patients with a recent HF hospitalization (in previous six months).  ^b^ Add HR for HF hospitalization or CV death of intended therapy to predict on-treatment risk/survival (methodology described in Supplementary Methods). Suggested HRs of risk-modifying therapies are shown in Table S5. For combinations of treatments, use the product of the individual HRs. | |

b. *Men*

| **Linear predictor (LP) for HF hospitalization or CV death**  LP_HFH+CV death_ = | | |
| --- | --- | --- |
|  | | 0.6767350532 (if first-year adjustment^a^) + 0.0457477942*(age_baseline – 40) + 1.8095133714 (if diabetes mellitus) + 0.1716017798 (if current smoker) + 0.2512267882*(ln(NT-proBNP) – ln(1250)) - 0.3829380460*(BMI – 25) + 0.0114835523*(BMI^2 – 25^2) - 0.0001044225*(BMI^3 – 25^3) + 0.0343981661*(heartrate – 80) - 0.0001983146*(heartrate^2 – 80^2) - 0.0215315853*(eGFR – 65) + 0.0001617259*(eGFR^2 – 65^2) - 0.1787339966*(Hb – 8) + 0.0061575399*(Hb^2 – 8^2) + 0.2893729126 (if NYHA class III or IV) + 0.2815851231 (if COPD) + 0.3225854802 (if previously hospitalized for HF) + 0.1423570674 (if ischemic heart disease) + 0.1507078243 (if atrial fibrillation) - 0.0211031719*age_vector (if diabetes mellitus) - ln(1.002295) + ln(cause-specific hazard ratio of risk-modifying therapy)^b^ |
| **Linear predictor (LP) for non-CV death**  LP_non-CV death_ = | | |
|  | | 0.23080253030 (if first-year adjustment^a^) - 0.03468837243*(age_baseline – 40) + 0.22736100293 (if diabetes mellitus) + 0.25986173405 (if current smoker) + 0.17940598574*(ln(NT-proBNP) – ln(1250)) - 0.74924785030*(BMI – 25) + 0.02127465160*(BMI^2 – 25^2) - 0.00019593651*(BMI^3 – 25^3) + 0.02151364444*(heartrate – 80) - 0.00009378628*(heartrate^2 – 80^2) - 0.03923164143*(eGFR – 65) + 0.00035508272*(eGFR^2 – 65^2) - 0.35862433394*(Hb – 8) + 0.01268086278*(Hb^2 – 8^2) + 0.25793839765 (if NYHA class III or IV) + 0.56385822436 (if COPD) + 0.20310848262 (if previously hospitalized for HF) - 0.15051652463 (if ischemic heart disease) - 0.11082222298 (if atrial fibrillation) - ln(1.232154) |
|  | Age_baseline in years at moment of prediction; age_vector is a sequence of ages (in rounded 3-month intervals) from the patient’s current age up to 89.75 years; NT-proBNP in pg/mL; BMI in kg/m^2^; heartrate in bpm; eGFR: estimated glomerular filtration rate in mL/min/1.73 m^2^; Hb (haemoglobin) in mmol/L.  ^a^ Add a first-year adjustment for the first four time intervals (3 months * 4 = 1 year) for patients with a recent HF hospitalization (in previous six months).  ^b^ Add HR for HF hospitalization or CV death of intended therapy to predict on-treatment risk/survival (methodology described in Supplementary Methods). Suggested HRs of risk-modifying therapies are shown in Table S5. For combinations of treatments, use the product of the individual HRs. | |

## Table S9. Discrimination of the LIFE-Preserved model for both outcomes in subgroups

|  |  |  | HF hospitalization or CV death | |  | Non-CV death | |
| --- | --- | --- | --- | --- | --- | --- | --- |
|  |  | N* | Events | C-statistic (95% CI) |  | Events | C-statistic (95% CI) |
|  | **Internal validation** | 16,750 | 6,120 | 0.732 (0.723 – 0.741) |  | 2,070 | 0.689 (0.679 – 0.699) |
|  | Women | 8,228 | 3,104 | 0.722 (0.709 – 0.735) |  | 1,026 | 0.693 (0.678 – 0.707) |
|  | Men | 8,522 | 3,016 | 0.741 (0.729 – 0.753) |  | 1,044 | 0.684 (0.670 – 0.698) |
|  | In-patient | 8,054 | 4,050 | 0.681 (0.668 – 0.693) |  | 1,313 | 0.677 (0.663 – 0.691) |
|  | Out-patient | 8,696 | 2,070 | 0.760 (0.746 – 0.773) |  | 757 | 0.697 (0.682 – 0.712) |
| Trials | **Emperor-Preserved** | 3,928 | 475 | 0.743 (0.720 – 0.767) |  | 144 | 0.619 (0.592 – 0.645) |
|  | Women | 1,969 | 213 | 0.761 (0.727 – 0.795) |  | 65 | 0.623 (0.583 – 0.662) |
|  | Men | 1,959 | 262 | 0.729 (0.695 – 0.762) |  | 79 | 0.608 (0.571 – 0.644) |
|  | Empagliflozin group | 1,963 | 217 | 0.758 (0.724 – 0.791) |  | 83 | 0.639 (0.600 – 0.677) |
|  | Placebo group | 1,965 | 258 | 0.731 (0.697 – 0.765) |  | 61 | 0.594 (0.557 – 0.631) |
|  | Diabetes | 1,886 | 266 | 0.745 (0.712 – 0.778) |  | 78 | 0.682 (0.646 – 0.717) |
|  | No diabetes | 2,042 | 209 | 0.738 (0.703 – 0.773) |  | 66 | 0.708 (0.671 – 0.746) |
|  | Obesity^a^ | 1,878 | 221 | 0.741 (0.706 – 0.777) |  | 65 | 0.697 (0.658 – 0.736) |
|  | No obesity | 2,050 | 254 | 0.749 (0.716 – 0.781) |  | 79 | 0.705 (0.672 – 0.739) |
|  | **TOPCAT-Americas** | 1,495 | 407 | 0.681 (0.642 – 0.719) |  | 70 | 0.673 (0.634 – 0.712) |
|  | Women | 768 | 196 | 0.679 (0.624 – 0.734) |  | 34 | 0.649 (0.591 – 0.706) |
|  | Men | 727 | 211 | 0.682 (0.629 – 0.735) |  | 36 | 0.704 (0.649 – 0.760) |
| Registries | **VA** | 4,499 | 1,675 | 0.687 (0.642 – 0.732) |  | 325 | 0.619 (0.540 – 0.699) |
|  | Men | 4,438 | 1,654 | 0.690 (0.645 – 0.735) |  | 322 | 0.616 (0.536 – 0.696) |
|  | **NHSE SDE** | 15,795 | 5,525 | 0.604 (0.593 – 0.616) |  | 2,570 | 0.621 (0.610 – 0.632) |
|  | Women | 8,485 | 3,025 | 0.601 (0.586 – 0.616) |  | 1,395 | 0.610 (0.595 – 0.624) |
|  | Men | 7,310 | 2,500 | 0.608 (0.591 – 0.624) |  | 1,175 | 0.635 (0.619 – 0.650) |
|  | **HF-Particles** | 838 | 254 | 0.693 (0.651 – 0.735) |  | 83 | 0.651 (0.605 – 0.697) |
|  | Women | 420 | 146 | 0.728 (0.672 – 0.784) |  | 32 | 0.558 (0.493 – 0.622) |
|  | Men | 418 | 108 | 0.653 (0.590 – 0.717) |  | 51 | 0.696 (0.620 – 0.772) |
| Harrell’s c-statistic for discrimination of the LIFE-Preserved model in derivation and validation cohorts. C-statistics for both outcomes (HF hospitalization or CV death and non-CV death) are displayed. ^a^ Obesity was defined as body mass index ≥ 30 kg/m^2^. * C-statistics are demonstrated for predicted risks at the 75th percentile of follow-up duration (5-year risks for SwedeHF, 2-year risks for EMPEROR-Preserved and NHSE SDE, 4-year risks for TOPCAT-Americas, 8-year risks for HF-Particles) and 10-year risks for VA . The number of women in VA was too low for estimating model performance separately for women (n = 62). As risks can only be calculated until age 90, patients for were removed from this analysis if their risk could not be calculated (e.g. 5 year risk for a 88 year old patient cannot be calculated). Therefore, number of patients can differ from those stated in the manuscript. Abbreviations: CI = confidence interval; HF = heart failure; CV = cardiovascular. | | | | | | | |

## Table S10. C-statistics of LIFE-Preserved compared to PREDICT-HFpEF in EMPEROR-Preserved

|  |  |  | ***HF hospitalization or CV death*** | |
| --- | --- | --- | --- | --- |
| **Observations** | **Events** | **Model** | **C-statistics (95% CI)** | **Difference (95% CI)** |
| 3,928 | 475 | LIFE-Preserved | 0.743 (0.720 – 0.767) | *ref* |
| 3,928 | 475 | PREDICT-HFpEF | 0.730 (0.705 – 0.756) | -0.013 (-0.028 – 0.002) |
| C-statistics were compared for 2 year risks of HF hospitalization or CV death in EMPEROR-Preserved and were calculated using the Score() function (contrasts = TRUE). Risks for PREDICT-HFpEF were calculated with the reported hazard ratios in their published article.^11^ History of TIA/stroke was not available in EMPEROR-Preserved data and the proportion of this predictor in the derivation data of PREDICT-HFpEF was used (0.123). | | | | |

## Table S11. EO ratios in external validation prior to recalibration

|  | **EO ratios ± SD** | | | |
| --- | --- | --- | --- | --- |
|  | HF hospitalization or CV death | | non-CV death | |
|  | Women | Men | Women | Men |
| EMPEROR-Preserved | 1.55 ± 0.008 | 1.31 ± 0.008 | 1.49 ± 0.005 | 1.38 ± 0.005 |
| TOPCAT-Americas | 1.03 ± 0.021 | 0.95 ± 0.022 | 1.54 ± 0.013 | 1.68 ± 0.012 |
| NHSE SDE | 1.24 ± 0.005 | 1.28 ±0.006 | 0.77 ± 0.004 | 0.79 ± 0.004 |
| HF-Particles | 1.05 ± 0.024 | 1.34 ± 0.023 | 2.36 ± 0.013 | 1.54 ± 0.017 |
| VA |  | 1.03 ± 0.013 |  | 1.97 ± 0.007 |
| EO ratios are calculated by dividing the sex-specific mean predicted risk by the observed cumulative incidence in study populations (details in Supplementary Methods). They are calculated at the following prediction horizon: 2-year (EMPEROR-Preserved and the NHSE SDE), 4-year (TOPCAT-Americas), 8-year (HF-Particles), 10-year (VA). The number of women in VA was too low for estimating model performance separately for women (n = 62). Abbreviations: EO ratio = expected-observed ratio; SD = standard deviation; HF = heart failure; CV = cardiovascular. | | | | |

# Supplementary Figures

## Figure S1. Flowchart of study population in SwedeHF Registry

Selection process of SwedeHF registrations before the first step in this flowchart can be found [here](https://kiheartfailure.github.io/shfdb4/population/).

^a^ February 1^st^ 2020 was used as the date for the start of the COVID-19 pandemic.

^b^ Patients with improved LVEF were defined as those with a previously recorded LVEF of <50% prior to baseline.

Abbreviations: HFpEF = heart failure with preserved ejection fraction; LVEF = left ventricular ejection fraction.

## Figure S2. Review of predictors in earlier models

The x-axis depicts al predictors that were included as candidate or final predictors in one of the models on the y-axis. The predictors are ordered based on their occurrences in the existing models (from left to right). The green dots represent final predictors of that specific model, the red dots represent candidate predictors of that specific model and the X represents that the predictor was not selected as candidate nor final predictor. Selection of predictors for LIFE-Preserved was based on this figure and included predictors that were two or more times used as final predictor in different models (left side of vertical line). The review was performed in September 2024.

## Figure S3. Effect of additional predictors on model discrimination for HF hospitalization or CV death

Extended models with each of the five additional predictors were derived in the SwedeHF Registry. Delta C-statistic between the extended models and the model with the core predictors was calculated using the Score() function from the *riskRegression* package.

Abbreviations: HF = heart failure.

## Figure S4. Functional form of transformed predictors and age interactions for primary outcome

Curves were generated by applying the estimated coefficients from the linear predictor (Table S8) across the observed range of each continuous variable. Hazard ratios were expressed relative to a prespecified reference value, which is indicated in the figure. This approach illustrates the shape and magnitude of the association between each predictor and the primary outcome, while accounting for nonlinear effects, including polynomial terms (BMI, heart rate, eGFR, haemoglobin) and log-transformed predictors (NT-proBNP), as well as interaction terms (e.g., age * diabetes mellitus). Hazard ratios were calculated on the log scale using the linear predictor, then exponentiated and plotted to show relative risks compared with the reference value.

a. *Women*

*b. Men*

## Figure S5. Smoothing of baseline survival

Presented plots show the 3-month baseline survivals across age for each outcome and for men and women separately (red dots). These estimates where smoothed using a LOESS function for the primary outcome and a Nonlinear Restricted Splines function for the competing outcome. Smoothed baseline survivals are presented by the blue line. The methodology is described in the Supplementary Methods section, and all baseline survivals after smoothing are provided in Table S8.

a. *Baseline hazards for women*

b. *Baseline hazards for men*

## Figure S6. Internal calibration in SwedeHF

a.*Short-term: 2-year risks*

Calibration of the LIFE-Preserved model in internal validation. Smoothed calibration plots of predicted risks versus observed risks. A histogram of the predicted risks is shown. Cohort sizes here may be lower than the total cohort population listed in Table 1 as risks can only be estimated up to age 90 (e.g., 89-year-olds can have 1-year risk but no risks for 2-year or higher and are consequently excluded at the horizon for validation). Abbreviations: HF = heart failure; CV = cardiovascular.

b.
*Long-term: 10-year risks*

Calibration of the LIFE-Preserved model in internal validation. Smoothed calibration plots of predicted risks versus observed risks. A histogram of the predicted risks is shown. Cohort sizes here may be lower than the total cohort population listed in Table 1 as risks can only be estimated up to age 90 (e.g., 89-year-olds can have 1-year risk but no risks for 2-year or higher and are consequently excluded at the horizon for validation). Abbreviations: HF = heart failure; CV = cardiovascular.

c.*Subgroup: healthcare setting (inpatient vs outpatient)*

Calibration of the LIFE-Preserved model in internal validation. Smoothed calibration plots of predicted risks versus observed risks. A histogram of the predicted risks is shown. Cohort sizes here may be lower than the total cohort population listed in Table S6 as risks can only be estimated up to age 90 (e.g., 89-year-olds can have 1-year risk but no risks for 2-year or higher and are consequently excluded at the horizon for validation). Abbreviations: HF = heart failure; CV = cardiovascular.

## Figure S7. C-statistics for HF hospitalization or CV death in sex-specific subgroups

Forest plot displaying the discrimination in all external validation data is based on Harrell’s C-statistic for HF hospitalization or CV death adjusted for non-CV death. Discrimination was assessed at the 75th percentile of follow-up duration (2-year risks for EMPEROR-Preserved and the NHSE SDE, 4-year risks for TOPCAT-Americas, 8-year risks for HF-Particles) and 10-year risks for VA. The number of women in VA was too low for estimating model performance separately for women (n = 62). Pooled C-statistics were calculated using inverse-variance weighting. Cohort sizes here may be lower than the total cohort population listed in Table 3 as risks can only be estimated up to age 90 (e.g., 89-year-olds can have 1-year risk but no risks for 2-year or higher and are consequently excluded at the horizon for validation). Abbreviations: CI = confidence interval.

## Figure S8. External calibration of HF hospitalization or CV death in women

Calibration of the LIFE-Preserved model for women in external validation. Risks were recalibrated in the NHSE SDE and EMPEROR-Preserved using EO-ratios specified in Table S11. The number of women in VA was too low for estimating model performance separately for women (n = 62). Smoothed calibration plots of predicted risks versus observed risks and a histogram of the predicted risks (in blue) is shown. Cohort sizes here may be lower than the total cohort population listed in Table 2 as risks can only be estimated up to age 90 (e.g., 89-year-olds can have 1-year risk but no risks for 2-year or higher and are consequently excluded at the horizon for validation). Abbreviations: HF = heart failure; CV = cardiovascular.

## Figure S9. External calibration of HF hospitalization or CV death in men

Calibration of the LIFE-Preserved model for men in external validation. Risks were recalibrated in the NHSE SDE and EMPEROR-Preserved using EO-ratios specified in Table S11. Smoothed calibration plots of predicted risks versus observed risks and a histogram of the predicted risks (in blue) is shown. Cohort sizes here may be lower than the total cohort population listed in Table 2 as risks can only be estimated up to age 90 (e.g., 89-year-olds can have 1-year risk but no risks for 2-year or higher and are consequently excluded at the horizon for validation). Abbreviations: HF = heart failure; CV = cardiovascular.

## Figure S10. External calibration of non-CV death

a. *Whole population*

b. *Women*

c.*Men*

Calibration for non-CV death of the LIFE-Preserved model in external validation for the whole population (a), women (b) and men (c). Risks were recalibrated in the NHSE SDE and EMPEROR-Preserved using EO-ratios specified in Table S11. The number of women in VA was too low for estimating model performance separately for women (n = 62). Smoothed calibration plots of predicted risks versus observed risks and a histogram of the predicted risks (in blue) is shown. Cohort sizes here may be lower than the total cohort population listed in Table 2 as risks can only be estimated up to age 90 (e.g., 89-year-olds can have 1-year risk but no risks for 2-year or higher and are consequently excluded at the horizon for validation). Abbreviations: HF = heart failure; CV = cardiovascular.

## Figure S11. External calibration of HF hospitalization or CV death in subgroups

*a. Stratified by treatment arm in EMPEROR-Preserved*

*b. Stratified by diabetes in EMPEROR-Preserved*

*c. Stratified by obesity in EMPEROR-Preserved*

Calibration of the LIFE-Preserved model in subgroups of EMPEROR-Preserved. Risks were recalibrated using EO-ratios specified in Table S11. For calibration stratified by treatment arm, the treatment effect of empagliflozin in the EMPEROR-Preserved trial was accounted for in risk prediction (HR = 0.79). Obesity was defined as body mass index ≥ 30 kg/m^2^. Smoothed calibration plots of predicted risks versus observed risks and a histogram of the predicted risks is shown. Cohort sizes here may be lower than the total cohort population listed in Table 2 as risks can only be estimated up to age 90 (e.g., 89-year-olds can have 1-year risk but no risks for 2-year or higher and are consequently excluded at the horizon for validation). Abbreviations: HF = heart failure; CV = cardiovascular.

## Figure S12. External calibration of LIFE-Preserved during COVID-19 period in the NHSE SDE

Calibration of both outcomes from the LIFE-Preserved model during the post COVID-19 period (main analysis) and during COVID-19 period (follow-up from Jan 1^st^ 2020 until March 1^st^ 2022) in the NHSE SDE. For this analysis, risks were not recalibrated in the NHSE SDE. Smoothed calibration plots of predicted risks versus observed risks and a histogram of the predicted risks is shown. Cohort sizes of the COVID cohort are lower than the cohort size of the main analysis as only patients with a HF hospitalization before Jan 1^st^ 2020 were included in this analysis (for more information on the identification of both cohorts in the NHSE SDE, see Supplementary Methods). Abbreviations: HF = heart failure; CV = cardiovascular.

## Figure S13. Descriptives for follow-up time for HF hospitalization or CV death in SwedeHF

**Figure S13a.** Boxplot of follow-up time for HF hospitalization or CV death in SwedeHF

**Figure S13b.** Unadjusted Kaplan-Meier survival curve for HF hospitalization or CV death in SwedeHF, stratified by sex

# Supplementary References

1.	Anker SD, Butler J, Filippatos G, Ferreira JP, Bocchi E, Böhm M, et al. Empagliflozin in Heart Failure with a Preserved Ejection Fraction. New England Journal of Medicine. 2021 Oct 14;385(16):1451–61.

2.	Pitt B, Pfeffer MA, Assmann SF, Boineau R, Anand IS, Claggett B, et al. Spironolactone for heart failure with preserved ejection fraction. New England Journal of Medicine. 2014 Apr 10;370(15):1383–92.

3.	Solomon SD, Claggett B, Lewis EF, Desai A, Anand I, Sweitzer NK, et al. Influence of ejection fraction on outcomes and efficacy of spironolactone in patients with heart failure with preserved ejection fraction. Eur Heart J. 2016 Feb 1;37(5):455–62.

4.	Kuźma Ł, Dąbrowski EJ, Kurasz A, Święczkowski M, Jemielita P, Kowalewski M, et al. Effect of air pollution exposure on risk of acute coronary syndromes in Poland: a nationwide population-based study (EP-PARTICLES study). The Lancet regional health Europe. 2024 Jun;41:100910.

5.	Deo S V, Reddy YN V, Zakeri R, Karnib M, Selvaganesan P, Elgudin Y, et al. Revascularization in ischaemic heart failure with preserved ejection fraction: a nationwide cohort study. Eur J Heart Fail. 2022 Aug;24(8):1427–38.

6.	Fletcher RA, Rockenschaub P, Neuen BL, Walter IJ, Conrad N, Mizani MA, et al. Contemporary epidemiology of hospitalised heart failure with reduced versus preserved ejection fraction in England: a retrospective, cohort study of whole-population electronic health records. Lancet Public Health. 2024 Nov;9(11):e871–85.

7.	Secure Data Environment - NHS England Digital [Internet]. [cited 2025 Dec 23]. Available from: https://digital.nhs.uk/services/secure-data-environment-service

8.	Home - British Heart Foundation - Data Science Centre [Internet]. [cited 2025 Dec 23]. Available from: https://bhfdatasciencecentre.org/

9.	Data Access Request Service (DARS) products and services - NHS England Digital [Internet]. [cited 2025 Dec 23]. Available from: https://digital.nhs.uk/services/data-access-request-service-dars/dars-products-and-services

10.	CVD-COVID-UK / COVID-IMPACT - British Heart Foundation - Data Science Centre [Internet]. [cited 2025 Dec 23]. Available from: https://bhfdatasciencecentre.org/areas/cvd-covid-uk-covid-impact/

11.	McDowell K, Kondo T, Talebi A, Teh K, Bachus E, de Boer RA, et al. Prognostic Models for Mortality and Morbidity in Heart Failure With Preserved Ejection Fraction. JAMA Cardiol. 2024 May 1;9(5):457–65.

12.	Pocock SJ, Ferreira JP, Packer M, Zannad F, Filippatos G, Kondo T, et al. Biomarker-driven prognostic models in chronic heart failure with preserved ejection fraction: the EMPEROR-Preserved trial. Eur J Heart Fail. 2022 Oct;24(10):1869–78.

13.	Kasahara S, Sakata Y, Nochioka K, Tay WT, Claggett BL, Abe R, et al. The 3A3B score: The simple risk score for heart failure with preserved ejection fraction - A report from the CHART-2 Study. Int J Cardiol. 2019 Jun 1;284:42–9.

14.	Rich JD, Burns J, Freed BH, Maurer MS, Burkhoff D, Shah SJ. Meta-Analysis Global Group in Chronic (MAGGIC) Heart Failure Risk Score: Validation of a Simple Tool for the Prediction of Morbidity and Mortality in Heart Failure With Preserved Ejection Fraction. J Am Heart Assoc. 2018 Oct 16;7(20):e009594.

15.	Komajda M, Carson PE, Hetzel S, McKelvie R, McMurray J, Ptaszynska A, et al. Factors associated with outcome in heart failure with preserved ejection fraction: findings from the Irbesartan in Heart Failure with Preserved Ejection Fraction Study (I-PRESERVE). Circ Heart Fail. 2011 Jan;4(1):27–35.

16.	Pocock SJ, Wang D, Pfeffer MA, Yusuf S, McMurray JJ V, Swedberg KB, et al. Predictors of mortality and morbidity in patients with chronic heart failure. Eur Heart J. 2006 Jan;27(1):65–75.

17.	Kontopantelis E, White IR, Sperrin M, Buchan I. Outcome-sensitive multiple imputation: a simulation study. BMC Med Res Methodol. 2017 Jan 9;17(1):2.

18.	Rubin DB. Multiple imputation for nonresponse in surveys. . New York: John Wiley & Sons; 1987.

19.	Kaasenbrood L, Bhatt DL, Dorresteijn JAN, Wilson PWF, D’Agostino RB, Massaro JM, et al. Estimated Life Expectancy Without Recurrent Cardiovascular Events in Patients With Vascular Disease: The SMART-REACH Model. J Am Heart Assoc. 2018 Aug 21;7(16):e009217.

20.	Dorresteijn JAN, Kaasenbrood L, Cook NR, van Kruijsdijk RCM, van der Graaf Y, Visseren FLJ, et al. How to translate clinical trial results into gain in healthy life expectancy for individual patients. BMJ. 2016 Mar 30;352:i1548.

21.	Berkelmans GFN, Gudbjörnsdottir S, Visseren FLJ, Wild SH, Franzen S, Chalmers J, et al. Prediction of individual life-years gained without cardiovascular events from lipid, blood pressure, glucose, and aspirin treatment based on data of more than 500 000 patients with Type 2 diabetes mellitus. Eur Heart J. 2019 Sep 7;40(34):2899–906.

22.	Jaspers NEM, Blaha MJ, Matsushita K, van der Schouw YT, Wareham NJ, Khaw KT, et al. Prediction of individualized lifetime benefit from cholesterol lowering, blood pressure lowering, antithrombotic therapy, and smoking cessation in apparently healthy people. Eur Heart J. 2020 Mar 14;41(11):1190–9.

23.	Brooke HL, Talbäck M, Hörnblad J, Johansson LA, Ludvigsson JF, Druid H, et al. The Swedish cause of death register. Eur J Epidemiol. 2017 Sep;32(9):765–73.

24.	Ludvigsson JF, Andersson E, Ekbom A, Feychting M, Kim JL, Reuterwall C, et al. External review and validation of the Swedish national inpatient register. BMC Public Health. 2011 Jun 9;11:450.

25.	Usman MS, Bhatt DL, Hameed I, Anker SD, Cheng AYY, Hernandez AF, et al. Effect of SGLT2 inhibitors on heart failure outcomes and cardiovascular death across the cardiometabolic disease spectrum: a systematic review and meta-analysis. Lancet Diabetes Endocrinol. 2024 Jul;12(7):447–61.

26.	Solomon SD, McMurray JJ V, Claggett B, de Boer RA, DeMets D, Hernandez AF, et al. Dapagliflozin in Heart Failure with Mildly Reduced or Preserved Ejection Fraction. New England Journal of Medicine. 2022 Sep 22;387(12):1089–98.

27.	Solomon SD, McMurray JJ V, Vaduganathan M, Claggett B, Jhund PS, Desai AS, et al. Finerenone in Heart Failure with Mildly Reduced or Preserved Ejection Fraction. N Engl J Med. 2024 Sep 1;

28.	Kosiborod MN, Deanfield J, Pratley R, Borlaug BA, Butler J, Davies MJ, et al. Semaglutide versus placebo in patients with heart failure and mildly reduced or preserved ejection fraction: a pooled analysis of the SELECT, FLOW, STEP-HFpEF, and STEP-HFpEF DM randomised trials. Lancet. 2024 Sep 7;404(10456):949–61.

29.	Packer M, Zile MR, Kramer CM, Baum SJ, Litwin SE, Menon V, et al. Tirzepatide for Heart Failure with Preserved Ejection Fraction and Obesity. N Engl J Med. 2025 Jan 30;392(5):427–37.
